# The Role of Health Kiosks: Scoping Review

**DOI:** 10.2196/26511

**Published:** 2022-03-29

**Authors:** Inocencio Daniel Maramba, Ray Jones, Daniela Austin, Katie Edwards, Edward Meinert, Arunangsu Chatterjee

**Affiliations:** 1 Centre for Health Technology University of Plymouth Plymouth United Kingdom

**Keywords:** kiosk, health systems, internet, review, online health information, telemonitoring, teleconsultation, consultation, telemedicine, behavior, promotion, health service, user experience, barrier, facilitator, remote consultation, mobile phone

## Abstract

**Background:**

Health kiosks are publicly accessible computing devices that provide access to services, including health information provision, clinical measurement collection, patient self–check-in, telemonitoring, and teleconsultation. Although the increase in internet access and ownership of smart personal devices could make kiosks redundant, recent reports have predicted that the market will continue to grow.

**Objective:**

We seek to clarify the current and future roles of health kiosks by investigating the settings, roles, and clinical domains in which kiosks are used; whether usability evaluations of health kiosks are being reported, and if so, what methods are being used; and what the barriers and facilitators are for the deployment of kiosks.

**Methods:**

We conducted a scoping review using a bibliographic search of Google Scholar, PubMed, and Web of Science databases for studies and other publications between January 2009 and June 2020. Eligible papers described the implementation as primary studies, systematic reviews, or news and feature articles. Additional reports were obtained by manual searching and querying the key informants. For each article, we abstracted settings, purposes, health domains, whether the kiosk was opportunistic or integrated with a clinical pathway, and whether the kiosk included usability testing. We then summarized the data in frequency tables.

**Results:**

A total of 141 articles were included, of which 134 (95%) were primary studies, and 7 (5%) were reviews. Approximately 47% (63/134) of the primary studies described kiosks in secondary care settings. Other settings included community (32/134, 23.9%), primary care (24/134, 17.9%), and pharmacies (8/134, 6%). The most common roles of the health kiosks were providing health information (47/134, 35.1%), taking clinical measurements (28/134, 20.9%), screening (17/134, 12.7%), telehealth (11/134, 8.2%), and patient registration (8/134, 6.0%). The 5 most frequent health domains were multiple conditions (33/134, 24.6%), HIV (10/134, 7.5%), hypertension (10/134, 7.5%), pediatric injuries (7/134, 5.2%), health and well-being (6/134, 4.5%), and drug monitoring (6/134, 4.5%). Kiosks were integrated into the clinical pathway in 70.1% (94/134) of studies, opportunistic kiosks accounted for 23.9% (32/134) of studies, and in 6% (8/134) of studies, kiosks were used in both. Usability evaluations of kiosks were reported in 20.1% (27/134) of papers. Barriers (e.g., use of expensive proprietary software) and enablers (e.g., handling of on-demand consultations) of deploying health kiosks were identified.

**Conclusions:**

Health kiosks still play a vital role in the health care system, including collecting clinical measurements and providing access to web-based health services and information to those with little or no digital literacy skills and others without personal internet access. We identified research gaps, such as training needs for teleconsultations and scant reporting on usability evaluation methods.

## Introduction

### Rationale

Health kiosks are publicly accessible computing devices used to provide access to a variety of services in the health care system. In a 2009 review, Jones [1] classified health kiosks as (1) opportunistic, placed in locations and waiting for use, and (2) integrated, designed into the clinical process. Seven possible roles for health kiosks were identified: taking medical histories, health promotion, self-assessment, consumer feedback, patient registration, patient access to records, and remote consultations.

At that time, 65% of households in the United Kingdom had internet access. By 2020, internet access had increased to 96% of households, most (98%) with a fixed broadband connection and 64% of households having internet access through mobile devices [2]. Older people have started to close the digital gap with younger age groups: recent internet use (the preceding 3 months) increased from 52% to 83% among individuals aged 65 to 74 years and from 20% to 47% among adults aged ≥75 years from between 2011 and 2019 [3]. Smartphone and tablet ownership in the United Kingdom has increased from 26% and 2% in 2011 to 78% and 58% in 2018, respectively [4]. These trends were also reflected worldwide. For example, 318,000 health-related apps for smartphones and tablets were listed in the app stores as of 2019 [5].

Data from the International Telecommunication Union show that these trends are reflected worldwide:

- The percentage of the world population with access to the internet increased from 26% (1.8 billion people) in 2009 to 51% (4 billion people) in 2019, broken down regionally as follows: 7% to 6% to 28.6% in Africa, 20.6% to 54.6% in the Arab States, 19% to 44.5% in Asia and the Pacific, 24.3% to 72.8% in the Commonwealth of Independent States, 59.6% to 82.5% in Europe, and 46.3% to 76.7% in the Americas.
- The number of mobile phone subscriptions per 100 people worldwide increased from 68 in 2009 to 107.8 in 2019 (meaning that in 2019 some people had more than one subscription).
- Fixed broadband connections per 100 people worldwide increased from 6.9 in 2009 to 14.8 in 2019. [6].

However, these developments do not make health kiosks redundant.

Despite these trends, various authors predict continued and even growing use of kiosks. Chen [7] has predicted that telehealth kiosks will be widespread by 2023. Similarly, a recent blog piece by Kochelek [8] has stated that health kiosks will be essential in the changing medical landscape for the following reasons: (1) kiosks will streamline patient check-in; (2) human-to-human contact will be minimized by the use of kiosks, which is vital during the coronavirus pandemic; and (3) telehealth kiosks placed in private areas of strategic locations will provide access to patient care for the public, and kiosks in group homes can also provide care for individuals who are immune compromised, reducing the need for travel and the risk of exposure. Thus, in contrast to expectations of the death of kiosks because of the use of mobile technologies, an alternative view is that health kiosks will still be a major part of the digital health landscape in the foreseeable future.

With the above in mind, we saw the need to investigate the evolution of the roles of health kiosks in the past decade and what possible roles they may play in the future. We were aware that there may have been reviews of health kiosks published since the work of Jones [1] in 2009, and an investigation by one of the authors revealed that the latest review before starting this one was published in 2013. As there have been great changes technologically in the past 7 years, the authors believed conducting a new review of the literature about health kiosks was justified.

### Background

#### Health Kiosks Versus Personal Smart Devices

As mentioned previously, the roles played by kiosks a decade ago may now be performed by personal smart devices (smartphones and tablets), especially in the delivery of health information. In 2019, 79% of adults (aged ≥18 years) in the United Kingdom owned a smartphone, and tablet ownership was estimated at 58%. However, this is subject to age differences, as only 40% of adults aged ≥65 years own smartphones [9,10]. Thus, health kiosks still play a role in providing access to health services to this segment of the population.

Even for smartphone and tablet owners, health information delivery via kiosks may still be useful as the information can be tailored, vetted, and delivered at the point of service. Although this may also be possible through smartphone apps, the app would need to be properly accredited and evaluated for accuracy, and the user would need to download it to their phone for it to be useful. However, tailored and vetted information delivered by a kiosk is already available without any further action on the part of the user.

The collection of clinical measurements is where health kiosks currently outperform personal smart devices. Although there are clinical measurement devices that can be connected to smartphones and tablets, such as blood pressure (BP) monitors, heart rate trackers, and glucose monitors, they have not yet become widespread in use. Health kiosks with linked measurement devices, such as stethoscopes, otoscopes, dermatoscopes, pulse oximeters, and BP monitors, can collect clinical data for telemonitoring or synchronous teleconsultations.

#### Health Kiosks for Remote Consultation

#### Overview

Teleconsultations are now also possible on smart devices or PCs without the need for a health kiosk. As reported in the news, during the lockdown period caused by the COVID-19 pandemic of 2020, only 7 of 100 general practitioner (GP) consultations were performed face to face, with the rest being done remotely. However, it is interesting to note that most of these consultations were still being conducted through telephone or text [11,12]. The news article also stated that there were still situations where patients needed to attend a practice in person, such as when BP or oxygen saturation needed to be read. These readings can be obtained using a properly equipped health kiosk. In a way, this is analogous to the existing situation of web-based banking apps and cash machines. The availability of web-based banking has not done away with the need for cash points, and banks have not yet relegated them to the scrap heap. Although web-based consultations can be facilitated through mobile devices, a substantial number of such encounters will require some physical examination or measurement using diagnostic instruments, which health kiosks can provide in lieu of face-to-face consultations. Manufacturers now provide solutions where health kiosks could be reconceptualized as *health pods*, similar to *photo booths*, with a private space to have consultations along with a range of devices performing point-of-care clinical measurements (eg, BP monitors, pulse oximeters, and stethoscopes). This is particularly useful in rural, remote, and deprived communities. There are several telehealth kiosk products currently on offer that follow this model. Some examples of these are the kiosks offered by MedicSpot, Amwell, RPM Solutions, and H4D.

MedicSpot is a web-based GP service in the United Kingdom that allows patients to connect to a physician via kiosks placed in pharmacies. It is available at ≥300 locations across the United Kingdom. The kiosk is available for walk-in consultations without appointments and contains medical equipment for examinations. The service provides patients access to a connected stethoscope; pulse oximeter; BP monitor; contactless thermometer; and an inspection camera to check the ear, nose, and throat. This is a private service that charges £39 (US $51.70) per consultation. MedicSpot has recently partnered with the British supermarket chain Asda to offer in-store GP video consultations with diagnostics [13-16].

The kiosk line of Amwell, which is based in Massachusetts, United States, comprises a fully enclosed kiosk model, freestanding open console kiosk, and tabletop kiosk model. All models include a touchscreen interface, integrated camera, credit card reader, handset for private audio, and sanitation features. They can be equipped with biometric and clinical measurement devices that allow virtual monitoring of a patient’s vital signs in real time. These include stethoscopes, otoscopes, pulse oximeters, BP cuffs, dermatoscopes, and thermometers [17]. Signs of Amwell’s growing strength in the telehealth market include a report that the company would be going public later in 2020, as well as raising US $194 million in funding by May 2020 [18].

Meanwhile, H4D, a health technology start-up based in Paris, France, completed a €15 million (US$ 16.4 million) round of funding in June 2020. H4D developed a telemedicine platform centered on the Consult Station, which is a connected telemedicine booth. It comprises all the necessary instruments and sensors for physicians to consult with patients via videoconference. The Consult Station has been deployed to ensure continuity of care and treatment for patients who are chronically ill and cannot be safely treated in traditional health care facilities.

It is worth noting that the abovementioned implementations were all in the private health sectors of the United Kingdom, the United States, and France. The adoption of health kiosks for teleconsultation by government-run health systems has been slow because of the strict rules for suppliers of equipment. Publicly funded health systems require evidence from numerous trials before adopting new technologies.

#### Health Kiosks for Responding to the COVID-19 Pandemic

Health authorities such as the World Health Organization and the Centers for Disease Control and Prevention have strongly urged ways of minimizing physical contact between patients and health care providers, otherwise known as *medical distancing*. Telehealth services are rapidly becoming one of the primary methods of reducing health care–related COVID-19 transmissions and protecting health personnel [19]. Telehealth kiosks equipped with monitoring and clinical measurement devices will allow comprehensive medical examination of the patient while maintaining medical distancing. The need for medical distancing is one of the drivers of the increased adoption of telemedicine kiosks.

In response to the COVID-19 pandemic, Elephant Kiosks (Cornwall, United Kingdom) introduced the COVID-19 Reception Kiosk, which offers the first point of contact for visitors and staff in workplaces, care homes, schools, and other public places. It offers an integrated contactless temperature check, a COVID-19 questionnaire, and email alerts to managers or the reception. It meets the infection control guidance and can be used to support contact tracing [20].

The H4d Consult Station has also been used to support hospitals during the COVID-19 pandemic, notably the Ramsay Health Vert-Galant Hospital’s emergency department (ED). The station was used to provide an initial screen and detect suspected COVID-19 cases. Using the Consult Station, the hospital was able to substantially reduce nurses’ intake time and protect them from the virus [21,22].

#### Health Kiosks for Remote and Rural Locations

One of the benefits of telehealth kiosks is making medical and specialist care available to remote places that medical professionals rarely visit. These places can be remote rural areas with poor infrastructure in countries such as India and Canada [23-25] or geographically remote places such as island communities or offshore installations, such as Scotland [26].

In their study, Nachum et al [27] found that in the United States, those who used teleconsultation kiosks were significantly more likely to be visitors to the area rather than local people, suggesting that a visit to the kiosk represented an opportunity to access care when not familiar with local services. This could suggest that the implementation of kiosks in areas experiencing high levels of tourism could help with their impact on health care provision. For example, the remote region of Cornwall, located on the southwesterly peninsula of the United Kingdom, sees as many as 4 million tourism trips each year, predominantly in the summer, putting huge pressure on infrastructure, including health care services [28]. In 2004, the estimated cost of the provision of primary health care to nonresidents in Cornwall was £4.7 million (US $6.23 million) [29]. The deployment of teleconsultation kiosks to cater to nonresidents could ease the pressure on local health services, especially if less urgent conditions could be managed by an autonomous mode of operation, with more urgent cases being seen *live* by a remote health care professional.

### Objective

To clarify how the role of health kiosks has evolved in the past decade and what roles they may play in the future, we conducted a scoping review. The primary objectives of this review are to describe the scope of kiosk use in health care (by patients, health care providers, or the general public), examine the roles played by health kiosks in the health care system, and investigate the barriers to and facilitators of the deployment of kiosks. We have developed the following research questions to address these objectives:

- What are the settings and health domains in which health kiosks are deployed, and what health services are they delivering?
- Are health kiosk interventions evaluated for usability, which has been identified as being important for effective digital health [30-32]?
- Finally, what are the barriers to and facilitators of the deployment of kiosks, especially for teleconsultation (eg, resources, infrastructure, and training [33])?

## Methods

### Overview

A scoping review is defined as a type of research synthesis that aims to “map the literature on a particular topic or research area” [34,35]. We undertook a scoping review of the published literature, as well as the gray literature available from websites and social media. To ensure that this was comprehensive, we also identified key informants from the contacts database of the Ehealth Productivity and Innovation in Cornwall and the Isles of Scilly Project [36], and through a Google search, we gathered information from them via emails and video calls.

### Definition of Health Kiosk

Computerized health kiosks have been defined as “freestanding units containing computer programs that provide users with information or services.” [37]. For this review, we used the following definition of health kiosks: public access computing devices providing or collecting information at any point in the health care journey. Kiosks are normally owned by a health service provider but used by various members of the public. Health application software (*apps*) were only included if they were made available on a public access device; if they were installed on personally owned devices such as smartphones, tablets, laptops, and desktop computers, they were excluded.

### Study Eligibility

Articles were included if they met the following criteria:

- Were about an actual implementation of a health kiosk and not a specification or nonfunctional prototype
- Were published in peer-reviewed publications, trade publications, and web-based health information technology publications
- Were published in the English language
- Were published between January 1, 2009, and June 1, 2020; we chose this period to update the previous review by Jones [1], which was published in 2009

Articles were excluded if they were design proposals for kiosks or nonfunctioning prototypes, if the device was a personal smart device rather than a publicly accessible device, or if they were in a language other than English.

### Information Sources and Search Terms

The first source was published in the literature. We searched three electronic literature databases: Web of Science, PubMed (including MEDLINE), and Google Scholar.

The primary search term was *health kiosk*, which we used for all 3 databases. We trialed using the search terms*[health]* *AND [kiosk] AND* *[touchscreen]*

As used in previous reviews, this resulted in the inclusion of papers mostly about personal smart devices such as smartphones and tablets, which we did not classify as kiosks.

The final search terms were as follows:

- PubMed:
  ((health[MeSH Terms] OR health[All Fields] OR health s[All Fields] OR “healthful”[All Fields] OR healthfulness[All Fields] OR healths[All Fields]) AND (kiosk[All Fields] OR kiosks[All Fields])) AND ((2009/1/1:2020/6/1[pdat]) AND (english[Filter]))
- Web of Science (advanced search):
  ALL=health AND ALL=kiosk
- Google Scholar (advanced search): exact phrase
  health kiosk
  anywhere in the article between 2009 and 2020

Gray literature and social media were also searched using the Google search engine for reports and publications on relevant websites, as well as the search function on two social media websites: Facebook and Twitter. Key informants (kiosk manufacturers) were identified through a Google search and the contact database of the Ehealth Productivity and Innovation in Cornwall and the Isles of Scilly Project; they were contacted via email and video calls. We asked the manufacturers about the use cases of their kiosk offerings, training needs for kiosk use, barriers and facilitators for successful deployment, and any relevant publications. A total of 3 kiosk manufacturers from around the world responded to our inquiries.

### Study Selection

**Figure 1:**
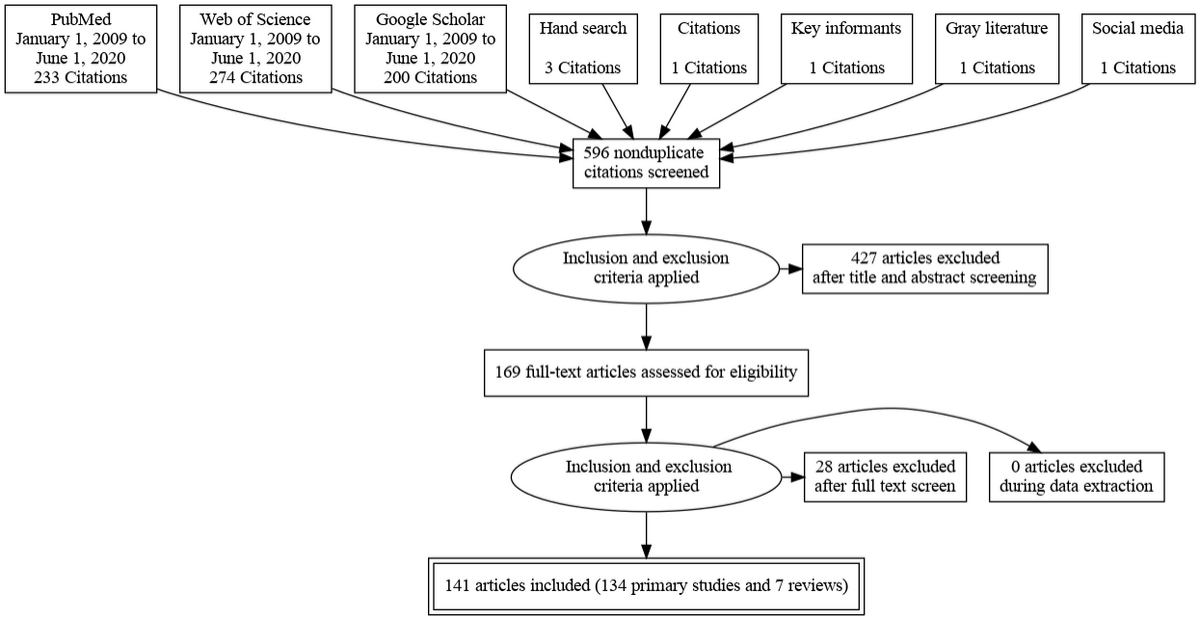
Diagram of articles reviewed for inclusion.

We collated citations from the literature search using the Mendeley (Elsevier) reference management software, and duplicate citations were eliminated. Author IDM screened the titles and abstracts to determine whether the study met the inclusion criteria. The studies were classified as either included or excluded. All articles classified as *included* had their full text retrieved for further review. DA, KE, and IDM then independently evaluated the full text of each study according to the agreed inclusion criteria. Disagreements were resolved by voting, with the third member serving as the tiebreaker. Critical appraisal was not performed as we did not compare study results, and streamlining of methods is acceptable in a scoping review [35]. We have presented the search results in a PRISMA (Preferred Reporting Items for Systematic Reviews and Meta-Analyses) flow diagram (Figure 1). A total of 141 articles met the inclusion criteria after a full-text review.

### Data Extraction and Analysis

A data extraction form was created based on the table of published studies on health kiosks used in the paper by Jones [1]. The extracted data items included the setting, number of kiosks, year of publication, country of implementation, type of access to the kiosk (opportunistic or referred), purpose of the kiosk, health conditions targeted by the kiosk, and whether and how the kiosk was evaluated for usability. Other significant information about the kiosk study was included as comments. DA, KE, and IDM performed the data extraction. The results were then encoded into a Microsoft Excel spreadsheet. IDM rechecked the data extraction table for consistency, with differences in coding resolved through discussions among IDM, DA, and KE. Frequency tables and graphs were constructed using R (version 4.2.0) [38].

## Results

### Overview

We present the results of our literature search as follows: (1) settings, purposes, and conditions addressed by the kiosks in the included papers; (2) country of publication; (3) year of publication; (4) type of kiosk access; (5) patient self–check-in kiosks; (6) reporting on the usability evaluation of kiosks; (7) telemonitoring and teleconsultation kiosks, training needs, and barriers to and enablers of adoption.

### Identified Publications

We identified 141 publications (Multimedia Appendix 1 [1,39-126]) by searching the PubMed (MEDLINE), Web of Science, and Google Scholar databases (Figure 1). All but 5% (7/141) were primary articles describing health kiosk implementations in clinical or community settings. Of the 7 systematic reviews, 3 (43%) were general reviews [1,39,40], 3 (43%) reviewed kiosks used for particular purposes (health information) [41-43], and 1 (14%) reviewed studies on kiosks used for BP monitoring [44]. The characteristics of the 141 included studies are summarized in Multimedia Appendix 1.

### Settings, Purposes, and Conditions

In the 134 primary studies, the most frequent setting (n=61, 47%) was secondary care, which was subdivided into specialty and outpatient clinics (n=34, 54%), EDs (n=26, 43%), and inpatient settings (n=5, 8%). The most frequently cited purpose (45/134, 33.6%) was providing health information (Table 1). Kiosk implementation most frequently targeted multiple health domains or conditions, followed by HIV. The setting *specialty clinics* included clinics such as sexual health and cancer clinics, where patients are referred from primary care and hospital department outpatient clinics. EDs are acute care centers, including accident and EDs within hospitals. Primary care settings included general practices, family medicine clinics, and community clinics. Community refers to the settings in which kiosks were deployed in nonclinical venues, including churches [45,46] and community centers [45,47]. *Multiple* refers to the implementation of kiosks in multiple categories; for example, in both a community pharmacy (retail outlet for medications and other health care–related products) and a library [48] or simultaneously in a nonclinical (eg, a social service agency, a church, a school, and a coffee shop) and a clinical (primary care clinic) setting [49].

**Table 1:** Summary of settings, purposes, and health domains for the primary studies (N=134).

Categories			Values, n (%)
**Settings**			
	**Secondary care**		63 (47)
		Specialty clinic	34 (54)
		Emergency department	26 (41)
		Hospital inpatient	5 (8)
	Community		32 (23.9)
	Primary care		24 (17.9)
	Pharmacy		8 (6)
	Multiple		7 (5.2)
**Purposes**			
	Health information		47 (35.1)
	Clinical measurements		28 (20.9)
	Screening		17 (12.7)
	Telehealth		11 (8.2)
	Patient registration		8 (6)
	Patient feedback		6 (4.5)
	Medication adherence		6 (4.5)
	Patient outcomes data		5 (3.7)
	Other		3 (2.2)
	Patient triage		3 (2.2)
**Health domains**			
	Multiple conditions		33 (24.6)
	HIV		10 (7.5)
	Hypertension		10 (7.5)
	Pediatric injuries		7 (5.2)
	Health and well-being		6 (4.5)
	Medication		6 (4.5)
	Cardiovascular disease		5 (3.7)
	Mental health		4 (3)
	Sexual health		4 (3)
	Acute care—emergency department		3 (2.2)
	Dementia		3 (2.2)
	Others		43 (32.1)

Table 2 shows the purposes of the kiosks arranged according to the setting. The most frequent purpose of kiosks in secondary care settings was health information, followed by screening and patient registration. In primary care settings, the most frequent purpose was likewise health information, followed by clinical measurements and medication adherence. This agrees with the findings of a review by Joshi and Trout [42], where most (58%) health information kiosks were found in clinical settings. The review concluded that health information kiosks were feasible mediums for disseminating health information among various users in clinical and community settings, particularly if computer-based tailoring is used.

**Table 2:** Purposes of the most frequent settings (N=134).

Purpose	Settings, n (%)						Total, n (%)
	Secondary care (n=63)	Community (n=32)	Primary care (n=24)	Pharmacy (n=8)	Multiple (n=7)		
Health information	23 (37)	7 (22)	12 (50)	1 (13)	4 (57)	47 (35.1)	
Clinical measurements	3 (5)	12 (28)	7 (29)	5 (63)	1 (14)	28 (20.9)	
Screening	12 (19)	4 (13)	1 (4)	0 (0)	0 (0)	17 (12.7)	
Telehealth	0 (0)	8 (25)	0 (0)	1 (13)	2 (29)	11 (8.2)	
Patient registration	7 (11)	0 (0)	1 (4)	0 (0)	0 (0)	8 (6)	
Patient feedback	5 (8)	0 (0)	1 (4)	0 (0)	0 (0)	6 (4.5)	
Patient outcomes data	6 (10)	0 (0)	0 (0)	0 (0)	0 (0)	6 (4.5)	
Medication reconciliation	3 (5)	0 (0)	2 (8)	0 (0)	0 (0)	5 (3)	
Other	1 (2)	1 (3)	0 (0)	1 (13)	0 (0)	3 (2.2)	
Patient triage	3 (5)	0 (0)	0 (0)	0 (0)	0 (0)	3 (2.2)	

For kiosks installed in community settings and retail pharmacies, the most frequent purpose was to collect clinical measurements.

For kiosks installed in specialty and outpatient clinics in secondary care, sexual health was the most frequent condition addressed by kiosks (4/134, 3%) [50-53], followed by breastfeeding (3/134, 2.2%) [54,55], cancer (2/134, 1.5%) [56,57], chronic kidney disease (2/134, 1.5%) [58,59], HIV (2/134, 1.5%) [60,61], mental health (2/134, 1.5%) [62,63], and orthopedics (2/134, 1.5%) [64,65], with other conditions making up the remaining implementations (12/134, 9%), as shown in Table 3.

In kiosks deployed in EDs, the most frequently encountered health domains were HIV, acute care, and asthma. The HIV screening process in the ED was streamlined using kiosks (7/134, 5.2%) [66,67]. The privacy and relative anonymity of HIV screening via kiosks are reasons cited for the successful deployment of kiosks for this purpose, as patients preferred screening via kiosks rather than by a person, possibly as they felt more secure disclosing intimate details to a computer screen than to a person [68,69]. Kiosks were also able to increase patient knowledge about HIV testing [60,61,70]. Other conditions that were screened using kiosks were dementia (3/134, 2.2%), mental health (2/134, 1.5%), domestic violence/home safety (2/134, 1.5%), alcohol and drug use (1/134, 0.7%), dermatology (1/134, 0.7%), and urinary tract infection (1/134, 0.7%). Dementia screening took place in kiosks deployed in community settings, as well as for dermatology, in which the kiosk was equipped to take images of skin lesions [71]. One of the mental health screening kiosks was set in primary care and the other in secondary care, and all other screening kiosks were deployed in secondary care, mostly in acute care/EDs. In the case of kiosks deployed in the community for screening, the situation is quite similar to asynchronous internet-based medical consultations.

Kiosks aided in the provision of acute care in the ED by performing patient triage, reliably collecting patient data, and significantly improving the time to identify new arrivals [72,73]. Other uses in the acute care pathway in the ED included patient registration [74] and medication adherence [75].

Primary care kiosks most frequently dealt with multiple conditions (7/134, 5.2%) [76-82], followed by cardiovascular disease (2/134, 1.5%) [83,84], general health and well-being (2/134, 1.5%) [85,86], hypertension (2/134, 1.5%) [87,88], and pediatric injuries (2/134, 1.5%) [89,90]. The community kiosks were for multiple conditions (8/134, 6%) and general health and well-being (2/134, 1.5%). Other conditions such as cardiovascular disease, dental health, dermatology, hypertension, infant mortality, pediatrics, and increasing social contact made up the rest (7/134, 5.2%). In studies where kiosks were deployed in pharmacies, the targeted health domains were hypertension [91-93], general health and well-being [94], and obesity [95]. In one of the studies, users accessed their personal health records through a kiosk at the pharmacy [96].

We examined the papers to determine if multiple papers evaluated the same kiosk system. A careful examination of the papers by authorship and system description revealed that 20.9% (28/134) of the primary studies were about 9 distinct kiosk systems. The 28 papers covered the settings, purposes, and conditions described in Table 4.

Thus, there were 115 distinct kiosk systems described in the 134 papers.

**Table 3:** Conditions and settings (N=134).

Condition	Setting, n (%)						Total, n (%)
	Secondary care (n=63)	Community (n=32)	Primary care (n=24)	Pharmacy (n=8)	Multiple (n=7)		
Multiple conditions	5 (8)	16 (50)	7 (29)	2 (25)	3 (43)	33 (24.6)	
HIV	10 (16)	0 (0)	0 (0)	0 (0)	0 (0)	10 (7.5)	
Hypertension	1 (2)	3 (9)	2 (8)	4 (50)	0 (0)	10 (7.5)	
Pediatric injuries	4 (6)	1 (3)	2 (8)	0 (0)	0 (0)	7 (5.2)	
Health and well-being	0 (0)	3 (9)	2 (8)	1 (13)	0 (0)	6 (4.5)	
Medication	4 (6)	0 (0)	2 (8)	0 (0)	0 (0)	6 (4.5)	
Cardiovascular disease	1 (2)	1 (3)	2 (8)	0 (0)	1 (14)	5 (3.7)	
Mental health	3 (5)	0 (0)	1 (4)	0 (0)	0 (0)	4 (3)	
Sexual health	4 (6)	0 (0)	0 (0)	0 (0)	0 (0)	4 (3)	
Acute care—ED^a^	3 (5)	0 (0)	0 (0)	0 (0)	0 (0)	3 (2.2)	
Breastfeeding	3 (5)	0 (0)	0 (0)	0 (0)	0 (0)	3 (2.2)	
Cancer	2 (3)	0 ()	1 (4)	0 (0)	0 (0)	3 (2.2)	
Dementia	0 (0)	3 (9)	0 (0)	0 (0)	0 (0)	3 (2.2)	
Pediatrics	2 (3)	1 (3)	0 (0)	0 (0)	0 (0)	3 (2.2)	
Smoking	1 (2)	0 (0)	1 (4)	0 (0)	1 (14)	3 (2.2)	
Asthma	2 (3)	0 (0)	0 (0)	0 (0)	0 (0)	2 (1.5)	
Chronic kidney disease	2 (3)	0 (0)	0 (0)	0 (0)	0 (0)	2 (1.5)	
Diabetes	0 (0)	0 (0)	0 (0)	0 (0)	2 (29)	2 (1.5)	
Domestic violence or home safety	2 (3)	0 (0)	0 (0)	0 (0)	0 (0)	2 (1.5)	
Obesity	0 (0)	0 (0)	1 (4)	1 (13)	0 (0)	2 (1.5)	
Orthopedics	2 (3)	0 (0)	0 (0)	0 (0)	0 (0)	2 (1.5)	
Alcohol and drug use	1 (2)	0 (0)	0 (0)	0 (0)	0 (0)	1 (0.7)	
Cervical cancer	0 (0)	0 (0)	1 (4)	0 (0)	0 (0)	1 (0.7)	
Childhood obesity	0 (0)	0 (0)	1 (4)	0 (0)	0 (0)	1 (0.7)	
Dental health	0 (0)	1 (3)	0 (0)	0 (0)	0 (0)	1 (0.7)	
Dermatology	0 (0)	1 (3)	0 (0)	0 (0)	0 (0)	1 (0.7)	
Dog bites	1 (2)	0 (0)	0 (0)	0 (0)	0 (0)	1 (0.7)	
Environmental health	1 (2)	0 (0)	0 (0)	0 (0)	0 (0)	1 (0.7)	
Food safety	1 (2)	0 (0)	0 (0)	0 (0)	0 (0)	1 (0.7)	
General medicine	1 (2)	0 (0)	0 (0)	0 (0)	0 (0)	1 (0.7)	
Genetic study	1 (2)	0 (0)	0 (0)	0 (0)	0 (0)	1 (0.7)	
Health care environment	1 (2)	0 (0)	0 (0)	0 (0)	0 (0)	1 (0.7)	
Infant mortality	0 (0)	1 (3)	0 (0)	0 (0)	0 (0)	1 (0.7)	
Organ donation	0 (0)	0 (0)	1 (4)	0 (0)	0 (0)	1 (0.7)	
Patient communication	1 (2)	0 (0)	0 (0)	0 (0)	0 (0)	1 (0.7)	
Radiology	1 (2)	0 (0)	0 (0)	0 (0)	0 (0)	1 (0.7)	
Rehabilitation	1 (2)	0 (0)	0 (0)	0 (0)	0 (0)	1 (0.7)	
Social contact	0 (0)	1 (3)	0 (0)	0 (0)	0 (0)	1 (0.7)	
UTI^b^	1 (2)	0 (0)	0 (0)	0 (0)	0 (0)	1 (0.7)	

^a^ED: emergency department.

^b^UTI: urinary tract infection.

**Table 4:** Kiosk systems described by multiple papers (N=28).

Kiosk system name	Country	Papers, n (%)	Settings	Purposes	Conditions
Telehealth Wellness Kiosk [97,98]	Unites States	2 (7)	Community	Telehealth	Multiple
HPV Project Kiosk [52,53]	Unites States	2 (7)	Secondary care	Health information	Sexual health
HIV Screening Kiosk [66-69,99-102]	Unites States	8 (29)	Secondary care	Screening	HIV
APHID Kiosk [103-106]	Unites States	4 (14)	Primary or secondary care	Medication adherence	Medication
PEMT Kiosk [54,55,107]	Unites States	3 (11)	Secondary care	Health information	Breastfeeding
My Kidney Care Centre [58,59]	Canada	2 (7)	Secondary care	Patient outcomes	Chronic kidney disease
KIO kiosk [108,109]	Unites States	3 (11)	Community	Screening or patient outcomes	Dementia
e-KISS kiosk [50,51]	Unites States	2 (7)	Secondary care	Health information	Sexual health
Safety in Seconds kiosk [110,111]	Unites States	2 (7)	Secondary care	Health information	Pediatric injuries

### Country of Kiosk Installation

The countries where the 115 kiosk systems were deployed and their corresponding settings are listed in Table 5.

**Table 5:** Countries and settings of included studies (N=134).

Country	Community (n=32), n (%)	Multiple (n=7), n (%)	Pharmacy (n=8), n (%)	Primary care (n=24), n (%)	Secondary care (n=63), n (%)	Total, n (%)
United States^a^	17 (53)	6 (86)	3 (38)	15 (63)	40 (63)	81 (60.4)
Canada^a^	0 (0)	0 (0)	2 (25)	0 (0)	3 (5)	5 (3.7)
United Kingdom^a^	2 (6)	0 (0)	1 (13)	2 (8)	1 (2)	6 (4.5)
Germany^a^	0 (0)	0 (0)	1 (13)	0 (0)	2 (3)	3 (2.2)
India^b^	2 (6)	0 (0)	0 (0)	0 (0)	1 (2)	3 (2.2)
South Korea^a^	1 (3)	0 (0)	0 (0)	0 (0)	2 (3)	3 (2.2)
New Zealand^a^	2 (6)	0 (0)	0 (0)	0 (0)	1 (2)	3 (2.2)
Portugal^a^	0 (0)	1 (14)	0 (0)	1 (4)	0 (0)	2 (1.5)
Singapore^a^	0 (0)	0 (0)	0 (0)	2 (8)	0 (0)	2 (1.5)
Australia^a^	1 (3)	0 (0)	0 (0)	0 (0)	0 (0)	1 (0.7)
Brazil^c^ and Portugal^a^	1 (3)	0 (0)	0 (0)	0 (0)	0 (0)	1 (0.7)
Japan^a^	1 (3)	0 (0)	0 (0)	0 (0)	0 (0)	1 (0.7)
Kenya^b^	1 (3)	0 (0)	0 (0)	0 (0)	0 (0)	1 (0.7)
The Philippines^b^	0 (0)	0 (0)	1 (13)	0 (0)	0 (0)	1 (0.7)
Sweden^a^	0 (0)	0 (0)	0 (0)	1 (4)	0 (0)	1 (0.7)
United States and Canada^a^	1 (3)	0 (0)	0 (0)	0 (0)	0 (0)	1 (0.7)

^a^High-income country.

^b^Lower middle–income country.

^c^Upper middle–income country.

Most kiosk studies were conducted in the United States, accounting for 70.4% (81/115) of the installed kiosk systems. Of the 115 installed kiosk systems, Canada and the United Kingdom had 5 (4.3%) and 6 (5.2%) systems, respectively, and Germany, India, South Korea, and New Zealand contributed 3 (2.6%) systems each. The list includes 11 high-income countries (Australia, Canada, Germany, Japan, New Zealand, Portugal, Singapore, South Korea, Sweden, the United Kingdom, and the United States), 1 upper middle–income country (Brazil), and 3 lower middle–income countries (India, Kenya, and the Philippines), as classified by the World Bank [112]. On the basis of the included primary studies, high-income countries had a higher proportion of kiosks situated in secondary care, whereas upper and lower middle–income countries had a greater proportion of kiosks deployed in primary care, the community, and pharmacies.

### Number of Studies Published Per Year

The studies included in the review were published in the period covering 2009 to 2020 (Table 6). The included 134 primary studies represent an almost 6-fold increase from the 25 studies cited by the review by Jones [1] published in 2009.

**Table 6:** Number of primary studies published per year from 2009 to 2020 (N=134).

Year	Studies, n (%)
2009	5 (3.7)
2010	10 (7.5)
2011	13 (9.7)
2012	8 (6)
2013	19 (14.2)
2014	17 (12.7)
2015	13 (9.7)
2016	11 (8.2)
2017	14 (10.4)
2018	12 (9)
2019	8 (6)
2020	4 (3)

### Type of Kiosk Access

Most (94/134, 70.1%) of the kiosks described in the included papers were integrated into clinical pathways (Table 7) and were cited mostly in secondary care (specialty clinics, EDs, and hospital inpatient clinics) and primary care facilities. The most common uses of these kiosks were delivering health information, clinical measurements, and screening. Opportunistic kiosks were described in approximately a quarter of the included studies and were most often found in community settings, clinical settings, and pharmacies. The most frequent uses of opportunistic kiosks were for delivering health information and taking clinical measurements.

**Table 7:** Type of access to health kiosk (N=134).

Setting	Type of access, n (%)				Total, n (%)
	Integrated (n=94)	Opportunistic (n=32)	Both (n=8)		
Secondary care	54 (57)	9 (28)	0 (0)	63 (47)	
Community	16 (17)	11 (34)	5 (63)	32 (23.9)	
Primary care	18 (19)	5 (16)	1 (13)	24 (17.9)	
Pharmacy	3 (3)	5 (16)	0 (0)	8 (6)	
Multiple	3 (3)	2 (6)	2 (25)	7 (5.2)	

### Patient Self–check-in Kiosks

One type of kiosk that has been widely deployed over the past decade is the patient self–check-in kiosk in general practices, outpatient clinics, and hospitals. In the United Kingdom, the rise of the electronic patient self–check-in kiosk can be traced to a guide released by the National Health Service (NHS) in 2009, entitled *Improving access, responding to patients: A “how-to” guide for GP practices.* The guide included a section on *self-service check-in screens*, which would allow patients to check themselves in for an appointment quickly [113]. The guide included practical tips on deployment, including estimated acquisition and maintenance costs. We could not find any official figures for the number of self–check-in kiosks in general practices and hospitals in the United Kingdom. The best data we could find was that a vendor of patient self-check-in software for GP surgeries estimated that their system had been used 30 million times since April 2018 [114]. Given that there are approximately 300 million GP appointments per year in the NHS [115], this vendor would account for 5% of patient appointments in the NHS in a 2-year period.

We found only a few studies in our literature search that evaluated patient self–check-in kiosks. These studies showed statistically significant reductions in waiting times for patients who checked in using the kiosks compared with those who did not [65,74]. What was surprising was the small number of studies in the published academic literature, given the growing adoption of patient self–check-in screens over the past 10 years. However, it may be that the studies were performed as service evaluations rather than academic research and not submitted for academic publication.

### Reporting on the Usability Evaluation of Kiosks

Of the 7 reviews retrieved, 3 (43%) mentioned usability as one of the outcomes reported in their included studies [40,42,43]. However, none of the reviews in the included literature mentioned the types of usability evaluation methods used in the included studies. Usability evaluations of health kiosks were reported in 20.1% (27/134) of the included primary studies, slightly higher than the 16% reported in a systematic review by Joshi and Trout [42].

The methods used for usability evaluation in 27 studies are listed in Table 8.

**Table 8:** Usability evaluations of health kiosks (N=27).

Methods	Values, n (%)
Questionnaires	13 (48)
Validated questionnaires	3 (11)
Focus groups	3 (11)
Interviews	7 (26)
Completion rates	4 (15)
Error rates	2 (7)
Multiple methods	10 (37)
Heuristic evaluation	3 (11)
Think-aloud	8 (22)
Click recording	2 (7)
Visual observation	5 (19)

Although questionnaires were the most frequently used usability evaluation method, only 11% (3/27) of studies used validated questionnaires, namely the System Usability Scale, the Technology Acceptance Model, and the Perceived Usefulness/Perceived Ease of Use questionnaire. Validated questionnaires enable researchers to compare their results with those of other studies. Questionnaires are subjective and quantitative methods. Some of the studies used qualitative methods such as focus groups, interviews, behavioral observations, and think-aloud sessions (8/27, 22%). Qualitative methods are usually used during the developmental stages. Objective methods were also used, such as completion times, error rates, and click recordings. Heuristic evaluation, using a checklist of desired heuristic features, was used only in a small minority of the studies (3/27, 11%). Approximately half of the studies (10/27, 37%) used >1 method of usability evaluation. Approximately 37% (10/27) of usability evaluations of health kiosks were able to identify usability issues. Most (16/27, 59%) reported that the users found the health kiosks easy to use.

### Telemonitoring Kiosks

Approximately 7.5% (10/134) of papers described the use of kiosks to deliver some form of telemonitoring or teleconsultations between 2011 and 2014. Most papers (6/10, 60%) described kiosks implemented in retirement communities for the use of older adults. Approximately 30% (3/10) of papers related to the same kiosk for a residential community of older adults in New Zealand [97,98,116]. Another kiosk was implemented in an urgent care pharmacy [27], and another, aimed at community-dwelling older adults, was tested in a laboratory setting [117]. One of the kiosks, not yet widely implemented, was deployed in a rural community health center [118].

Approximately 80% (8/10) of papers described 5 different kiosks that provided telemonitoring services, including monitoring of vital signs such as BP and oximetry. These kiosks included a screen but did not allow for 2-way live communication with a health care provider. Telemonitoring kiosks aimed at older adults often also included measures of cognitive performance and the opportunity for residents to engage with educational videos and *brain fitness* games. Health information collected by the kiosk was transmitted electronically to relevant health care professionals who could monitor ongoing conditions such as hypertension [119] and cognitive decline [120,121]. In some cases, users were also able to download their information and observe changes over time [97,98,116]. A kiosk designed for a rural community center in India, although not yet widely implemented, also included functions that enable the detection of malaria and tuberculosis and upload of radiology images [118].

### Teleconsultation Kiosks

Of the 20% (2/10) of papers that outlined kiosks that offered the opportunity for users to interact in a live 2-way consultation with a health care professional, one of them, HealthSpot, is no longer in operation. We will discuss the history of HealthSpot in greater detail in the following sections. The kiosk that is still in operation has been implemented in 7 urgent care pharmacies across New York City and included audiovisual equipment enabling a web-based consultation with an ED physician, a BP cuff, a pulse oximeter, and a thermometer [27]. This provider also offered the same service but via a mobile app. The authors reported that out of a total of 1996 web-based consultations conducted, only 238 were at kiosks, and the daily use of each kiosk location was low. However, people who used the kiosks were less likely to experience technical difficulties compared with those who used the app. Interestingly, the authors also found that those who used the kiosks were significantly more likely to be visitors to the area than local people, suggesting that a visit to the kiosk represented an opportunity to access care when not familiar with local services.

### Training Needs for Implementing Kiosks for Telemonitoring and Teleconsultations

Only 20% (2/10) of papers detailing kiosks providing telemonitoring or teleconsultation services described the training required to implement the kiosk. Wilamowska et al [116] briefly noted that the kiosk vendor organized 2 training sessions to familiarize the research team members with the design and details of the kiosk and its output data. Training for end users (older adults) was not described [116].

Resnick et al [119] described how their kiosk for older adults incorporated training for both researchers and end users [119]. Retirement center employees and researchers were first taught how to use the device by the kiosk developers. The research staff then trained older people on how to use the kiosk equipment. No further details on what the training involved were included in the paper. However, nearly all older adults reported being *very comfortable* with the technology; 81% reported that it was easy to use, and 98% reported that they would recommend it to others. However, analysis of compliance data revealed that kiosk use decreased over time, and the authors suggested that enhanced training on the use of equipment may facilitate the continued use of the kiosk following the initial *honeymoon* period.

### Barriers to and Enablers of Teleconsultation Kiosk Adoption

The experience of the telemedicine kiosk pioneer HealthSpot provides a good understanding of barriers to adoption. HealthSpot was founded in 2010 and raised approximately US $46.7 million in funding. It also attracted several big-name partners such as Xerox, MetroHealth, Mayo Health, Kaiser Permanente, the Cleveland Clinic, and Rite Aid (the third largest retail pharmacy chain in the United States). HealthSpot’s telemedicine kiosk was fully enclosed and used proprietary cloud-based software and was equipped with high-definition videoconferencing, a BP cuff, thermometer, stethoscope, otoscope, dermatoscope, and a built-in weighing scale [122]. Despite this promising start, HealthSpot ceased operations in December 2015.

Mudumba [123] and Chen [7] enumerated the following reasons for the failure of HealthSpot:

- Too much time spent on the academic validation of kiosk functionality rather than vetting the business model in the market
- Requires prescheduling appointments for HealthSpot kiosk users, which goes against the utility aspect of telehealth
- No integration with mobile health platforms
- Inadequate planning for scaling
- The target market was too small

The HealthSpot kiosk used proprietary videoconferencing software, whose high cost weakened the HealthSpot business model. According to Chen [7], kiosks need to cost <US $5000 per unit for the business model to succeed. These lessons must be considered when companies attempt to enter the telehealth kiosk market.

Some other studies also mentioned barriers to and enablers of kiosk adoption. Venkatesh [23] noted that advice from strong and weak ties was an enabler of kiosk adoption by mothers. Conversely, hindrance from strong and weak ties was a barrier to kiosk adoption [23]. Ackerman [124] investigated the reasons for nonadoption of a kiosk to screen for urinary tract infection in an ED setting. The kiosk had previously been successfully adopted in an urgent care clinic setting. The research showed that kiosk algorithms were not adaptable to changing situations in a busy emergency room. The researchers also failed to involve triage nurses in the development of the system, which resulted in disengagement and a nonsupportive attitude toward the kiosk.

## Discussion

### Principal Findings

In this review, we sought to describe the current roles that health kiosks play in the health care system in terms of settings, purposes, health domains, and type of kiosk (opportunistic or integrated into a care pathway), as reported in the existing literature. We also investigated the use of kiosks for patient self–check-in, the extent of reporting of the usability evaluation of health kiosks, and the factors that affect the use of kiosks for remote consultations. We identified that clinical settings still comprised most (87/134, 64.9%) sites for health kiosks, and community settings accounted for some (32/134, 23.9%) of the kiosk installations in the included studies. Retail pharmacy settings comprised 5.9% (8/134) of the included studies. However, BP kiosks have long been deployed in pharmacies for quite some time. In 2012, Alpert [91] reported that 1 million BP readings per day were recorded in BP kiosks in pharmacies. Currently, kiosks with more functions are being deployed in pharmacies, including drug dispensing [125], teleconsultation [13], drug disposal, and health measurements and information kiosks [126].

### Comments on the Findings

#### Country of Kiosk Installation, Clinical Integration, Increase in Publication, and Usability of Kiosks

When looking at the countries of installation, high-income countries dominate in the studies on health kiosks included in our review, accounting for 73% (11/15) of the countries where kiosks were installed. Regarding countries and settings, it can be noted that high-income countries have a larger proportion of kiosks in secondary care settings, whereas upper and lower middle–income countries tend to have their kiosks installed in community and primary care settings. This reflects the more advanced health infrastructure of high-income countries, which can afford to deploy information technology solutions in their health systems. A study on barriers to and facilitators of the deployment of health kiosks in Iran, an upper middle–income country, listed a lack of resources as one of the barriers [127]. A report from the World Health Organization / World Bank in 2017 stated that half of the world’s population still lacks access to essential health services [128]. In situations where health resources are in short supply, kiosks will probably not be high in the list of priorities.

Kiosks are more likely to be integrated into a clinical pathway (94/134, 70.1%), especially if they were in a clinical setting. Community kiosk installations were evenly divided between integrated access and opportunistic/dual access. In both clinical and community settings, health information and clinical measurements were the most frequent purposes for kiosks.

The 6-fold increase in publications on health kiosks is an indication of the growing use of computerized kiosks in health care. This also coincides with the increased growth of the computerized kiosk market in other sectors, such as retail, hospitality, and banking, during the same period [129]. However, there has been a drop in the number of publications per year since 2013, which could lead to the conclusion that there has been a decrease in the relevance and interest in health kiosks since that year. However, another explanation could be that because of the continued growth of the use of health kiosks and self-service kiosks in general since 2010, as stated in market research reports, the use of health kiosks has become more normalized since 2013, such that fewer researchers are publishing work in this area in the same way that there are few research papers about airline check-in kiosks and automated teller machines.

The proportion of health kiosk studies that include a usability evaluation of the kiosk has not changed much since 2014 and is in the minority (<20%). This is consistent with the low rate of reporting on usability evaluations of digital health technologies in general [31]. There is also a lack of use of validated questionnaires for usability evaluations, making comparisons of usability between studies difficult. As user experience evaluations are now required for the commissioning of new digital health devices [130], manufacturers who wish to enter and develop products in the growing health kiosk market will need guidance, capacity, and capability building in user experience evaluation.

#### Limitations and Strengths of the Review

This review has a few limitations. We were only able to search for papers published in English, which may have excluded several papers about health kiosks that were not published in English. This means that we were not able to include papers about kiosks installed in countries such as China, Japan, South Korea, and others if they were published in a language other than English. We were also constrained to reduce our search terms, as the use of the term *touchscreen* (as was done in the 2009 review by one of the authors) resulted in the inclusion of many papers on smartphones and tablets, which were clearly not kiosks. In addition, the term *kiosk* is not part of a controlled vocabulary (eg, Medical Subject Heading). We deliberately excluded papers on proposed kiosks, including only papers on actual kiosk installations. Some of these kiosk proposals may have become actual kiosks in the interim; however, we would have no way of knowing which one was successfully implemented. The quick pace of technological change also outstrips the pace of academic publishing; hence, we also included information gathered from web search engines and key informants. Finally, the competitive nature of digital health technology makes information about development methods closely guarded trade secrets, which makes the publication of these methods in academic journals unlikely.

We were aware that there were existing reviews on health kiosks before we started this scoping review. Our search identified 7 prior reviews, the latest of which was published in 2013. It was our consensus that in the 7 years since the last review, there were sufficient technological advances to warrant a new review. In the process of writing the findings of this review, a new systematic review of integrated health kiosks was published [131]. This review only covered publications up to 2018 and only included 37 articles. Our review covers publications from January 2009 to June 2020 and includes 137 articles. Thus, one of the strengths of our review is that it is more timely and comprehensive and complements the findings of previously published reviews.

#### Implications of the Findings

##### Kiosk Adoption: Barriers and Enablers and Training Needs

A recent qualitative study of 20 experts in Iran investigated their perceptions of the barriers to and facilitators of health kiosk adoption [127]. They identified lack of resources, low digital literacy, and resistance from health system officials as some of the barriers to adoption. On the other hand, high internet and electric power penetration rates, deployment of telemedicine, and integrated management of health services were cited as facilitators for adoption. The barriers to and enablers of kiosk adoption were mentioned in only a few of the studies included in our review; thus, there is a need for further research on this topic.

The current success of MedicSpot in the United Kingdom contrasts greatly with the failure of HealthSpot in the United States. MedicSpot follows the points made by Chen [7] and Mudumba [123] by allowing walk-in consultations, integrating with a mobile platform, and planning carefully for scaling. MedicSpot was shortlisted for the Digital Innovation Team of the Year at the 2019 British Medical Journal Awards [16].

This brings us to the need for training in using health kiosks for teleconsultation. Although most of the included papers about kiosks for telemonitoring and teleconsultations were aimed at older adults with less technical experience, it is surprising that end user training needs are not frequently described in more detail. It is possible that kiosk use with touch screens has been normalized in other areas of daily living (eg, banking and supermarket shopping), and thus, their use is seen to be intuitive. The lack of training may also reflect that, in some cases, a kiosk may be accompanied by a trained *person* to support the use and management of technical issues. This may be in accordance with previous NHS guidance that recent technology be introduced together with someone who can assist inexperienced users [113]. This is also being practiced in the Danish *chronic obstructive pulmonary disease briefcase* telemedicine intervention, where the patient’s equipment was installed by a technician who also provided instructions on how to switch the system on and off and how to position the finger clip pulse oximeter [132]. The learning needs of health professionals in using video calls to support patients have been successfully identified through workshops [133]. A similar methodology can be used to create training programs for health care professionals to use video calls for teleconsultations.

##### Patient Self–check-In Kiosks

The adoption of patient self–check-in kiosks has had its share of criticism and negative news reports. An opinion piece by Williamson [134] warned that the impersonality of these systems is contrary to general practice’s emphasis on personal and therapeutic relationships. Most practices have responded to this by still giving patients the option of checking in for their appointments via a human receptionist. There have also been concerns about the display of personal information on kiosk screens that could be viewed by others [135], as well as the hygiene implications of multiple users touching the same kiosk. The solutions to this are limiting the display of information to the appointment time, health care provider, and examining room and by providing hand sanitizing gel and regularly disinfecting the kiosk screen. Further research on no-touch interfaces with kiosks, such as voice and gestures, can also decrease the possibility of spreading infections [136,137]. Another news item in 2016 reported that some patients exaggerated their symptoms when answering questions at self–check-in kiosks installed in the accident and ED of a hospital to jump the queue. The hospital responded by combining the use of the electronic system with face-to-face input from senior clinicians to ensure that more accurate information was gathered [138]. This points to the need for a regular audit of the security and privacy of the health kiosk installation. Some research has been conducted on ensuring the security and privacy of kiosks [139]; however, more work will be needed as the number of health kiosk installations increases. Security is related to the need to regularly update kiosk software to respond to security threats, as well as to meet changing needs as health care situations evolve. A cloud-based platform may be a solution; however, it also creates the need for a constant connection to the internet. All these issues require further research.

### Conclusions

In conclusion, this review characterizes the present roles that health kiosks play in the health care system based on the existing literature. We have established that despite the growth in erstwhile health kiosk replacements such as personal smart devices and their attendant apps, health kiosks still have a vital role to play in the health care system, such as in the collection of clinical measurements for teleconsultations, provision of access to eHealth for the older population without smartphones, and provision of tailored and vetted health information at the point of service. We also identified research gaps such as identifying training needs for using the kiosk/video call combination for teleconsultations; methods for usability testing of kiosks; barriers to and enablers of kiosk deployment; and the exact extent of kiosk use for patient self–check-in for primary, secondary, and tertiary care. We also recommend the implementation of programs that will increase the capability and capacity of kiosk developers to perform user experience evaluations, both during development and while in service.

## Appendix

Multimedia Appendix 1 Summary of included studies.

Multimedia Appendix 2 PRISMA (Preferred Reporting Items for Systematic Reviews and Meta-Analyses) checklist.

## Abbreviations

BP: blood pressure
ED: emergency department
GP: general practitioner
NHS: National Health Service
PRISMA: Preferred Reporting Items for Systematic Reviews and Meta-Analyses
