# Supplementary material for: The Role of Health Kiosks: Scoping Review
Source: JMIR Med Inform. 2022 Mar 29;10(3):e26511. doi: 10.2196/26511 (PMC9006133; doi:10.2196/26511)
Supplement: Multimedia Appendix 1 [file medinform_v10i3e26511_app1.pdf]

Multimedia Appendix: Summary of Study Characteristics

Author	Journal	Title	Setting	# Kiosks	Yr	Country	Access	Comments	Purpose	Condition
Mahmood, Asos and Wyant, David K and Kedia, Satish and Ahn, SangNam and Powell, M Paige and Jiang, Yu and Bhuyan, Soumitra S	The Journal of emergency medicine	Self-Check-In Kiosks Utilization and Their Association With Wait Times in Emergency Departments in the United States.	Emergency Department	Unclear - 9% of US ED's are using a check-in Kiosk	2020	US	O	Secondary analysis of national data - wait time in ED's with kiosks was 56.8% shorter than those without	Patient registration	Acute Care - ED
Markowski, Marianne	Societies	The Teletalker � A Design Researcher's Tool to Explore Intergenerational Online Video Connectivity in-the-Wild	Community	2	2020	UK	O/R	Reactions/Attitudes towards the kiosk to inform design. Overall positive reactions from younger people, mixed from older. Volume button did not work as predicted	Communication	Social Contact
Mosher ZA, Hudson PW, Lee SR, Perez JL, Arguello AM, McGwin G, et al.	South Med J	Check-in Kiosks in the Outpatient Clinical Setting: Fad or the Future?	Specialty Clinic	6	2020	US	R	Six kiosks were implemented in a large academic orthopedic clinic. Kiosks offer to reduce check-in duration and increase POS revenue without negatively affecting patient satisfaction.	Patient registration	Orthopedics
Nachum S, Gogia K, Clark S, Hsu H, Sharma R, Greenwald PW	Telemed J E Health	An Evaluation of Kiosks for Direct-to-Consumer Telemedicine Using	Pharmacy	7	2020	US	O	Retrospective review of adult patients using pharmacy-based	Telehealth	Various

Author	Journal	Title	Setting	# Kiosks	Yr	Country	Access	Comments	Purpose	Condition
		the National Quality Forum Assessment Framework						kiosks vs app on personal device. Kiosk users were older, away from home, and had less technical difficulty. Accounted only for minority (12%) of DTC use.		
Coyle, Natalie and Kennedy, Andrew and Schull, Michael J and Kiss, Alex and Hefferon, Darren and Sinclair, Paul and Alsharafi, Zuhair	CJEM	The use of a self-check-in kiosk for early patient identification and queuing in the emergency department	Emergency Department	2	2019	CA	R	97% of people arriving in ED during peak hours used the kiosk. Kiosk significantly improved the time to identify new arrivals. Found no improvement in time-to-triage or tome-to MD.	Patient Triage	Acute Care - ED
Jung, Hee-Won and Roh, Hyunchul and Cho, Younggun and Jeong, Jinyong and Shin, Young-Sik and Lim, Jae-Young and Guralnik, Jack M and Park, Jihong	Journal of the American Geriatrics Society	Validation of a Multi-Sensor-Based Kiosk for Short Physical Performance Battery	Specialty Clinic	1	2019	KR	R	Prospective, cross sectional study: The kiosk proved to be a viable and efficient method for performing the Short Physical Performance Battery tests	Clinical Measurements	Rehabilitation
Kripalani, Sunil and Hart, Kimberly and Schaninger, Caitlin and Bracken, Stuart	American Journal of Health-System Pharmacy	Use of a tablet computer application to engage patients in updating their	Emergency Department	1	2019	US	R	Quasi-experimental study - tablet computer-based	Medication reconciliation	Medication

Author	Journal	Title	Setting	# Kiosks	Yr	Country	Access	Comments	Purpose	Condition
and Lindsell, Christopher and Boyington, Dane R		medication list						medication history application was feasibly to implement in a busy ED. Use was associated with more updated to EPHR medication list		
Niem{\"{o}}ller, Saskia and H{\"{u}}bner, Ursula and Egbert, Nicole and Babitsch, Birgit	Studies in health technology and informatics	How to Access Personal Health Records? Measuring the Intention to Use and the Perceived Usefulness of Two Different Technologies: A Randomized Controlled Study	Pharmacy	1	2019	DE	R	RCT kiosk vs app for accessing PHR. Differences in intention to use and perceived usefulness were examined. Both were equally acceptable for people of different genders, ages, and tech experience	Access PHR	Various
Sano, Mary and Zhu, Carolyn W and Kaye, Jeffrey and Mundt, James C and Hayes, Tamara L and Ferris, Steven and Thomas, Ronald G and Sun, Chung-Kai and Jiang, Yanxin and Donohue, Michael C and Schneider, Lon S and Egelko, Susan and Aisen, Paul S and	Alzheimer's {\&} Dementia	A randomized clinical trial to evaluate home-based assessment of people over 75 years old	Home	1	2019	US	R	RCT: mail +telephone questionnaire vs. automated telephone, vs. kiosk. Those in the kiosk arm were more likely to drop-out of the trial earlier. Staff resources were higher in the kiosk arm.	Cognitive assessments	Dementia

Author	Journal	Title	Setting	# Kiosks	Yr	Country	Access	Comments	Purpose	Condition
Feldman, Howard H								Low efficiency of kiosk-based assessment compared to live assessors		
Shafii, Taraneh and Benson, Samantha K and Morrison, Diane M and Hughes, James P and Golden, Matthew R and Holmes, King K	PLOS ONE	Results from e-KISS: electronic-KIOSK Intervention for Safer Sex: A pilot randomized controlled trial of an interactive computer-based intervention for sexual health in adolescents and young adults	Specialty Clinic	2	2019	US	R	Pilot RCT: two different types of information delivery, both via kiosk. One included video and personalized feedback from a physician avatar. Using the Kiosk was feasible in a clinic setting and was acceptable to users.	Health Information	Sexual Health
Tompson, Alice and Fleming, Susannah and Lee, Mei-Man and Monahan, Mark and Jowett, Sue and McCartney, David and Greenfield, Sheila and Heneghan, Carl and Ward, Alison and Hobbs, Richard and McManus, Richard J	BMJ Open	Mixed-methods feasibility study of blood pressure self-screening for hypertension detection	Primary Care	2	2019	UK	R	Feasibility study- no. of patients using the kiosk, cost, user feedback. Only 186/15624 used the kiosk to transfer BP into EPHR. Patients and HCP experienced tech difficulties hindering use. Was not cost effective due to limited use	Clinical Measurements	Hypertensi on
Young, Alexander S and Cohen, Amy N and Hamilton, Alison B and Hellemann,	The Journal of Behavioral Health Services {\&}	Implementing Patient-Reported Outcomes to Improve the Quality	Specialty Clinic	8	2019	US	R	Can PROMs support evidence based practice and improve care	Patient Outcomes Data	Mental Health

Author	Journal	Title	Setting	# Kiosks	Yr	Country	Access	Comments	Purpose	Condition
Gerhard and Reist, Christopher and Whelan, Fiona	Research	of Care for Weight of Patients with Schizophrenia						for schizophrenia? The kiosk was important from promoting awareness of wellness issues. Clinicians thought it was an easy way for pp's to monitor their progress.		
Abraham, Olufunmilola and Patel, Megha and Feathers, Alison	Health Services Research and Managerial Epidemiology	Acceptability of Health Kiosks Within African American Community Settings	Community	2	2018	US	O	study aimed to explore the acceptability, usability, usefulness, and overall satisfaction of health kiosks in African American majority community settings. study showed that health kiosks are accepted among African Americans in community settings such as churches and community centers. Participants found the kiosks easy to use and an overall useful tool to help	Health Information, Clinical measurements	Various

Author	Journal	Title	Setting	# Kiosks	Yr	Country	Access	Comments	Purpose	Condition
								manage their health.		
Boltin, Nicholas and Valdes, Diego and Culley, Joan M and Valafar, Homayoun	JMIR mHealth and uHealth	Mobile Decision Support Tool for Emergency Departments and Mass Casualty Incidents (EDIT): Initial Study	Emergency Department	1	2018	US	R	Utility assessment of EDIT system inc. kiosk component during a mock mass incident. The system demonstrated reliability when collecting patient data through a self-service kiosk, and thus reducing burden on hospital resource	Patient Triage	Acute Care - ED
Brinker, Titus Josef and Brieske, Christian Martin and Esser, Stefan and Klode, Joachim and Mons, Ute and Batra, Anil and R{\"{u}}ther, Tobias and Seeger, Werner and Enk, Alexander H and von Kalle, Christof and Berking, Carola and Heppt, Markus V and Gatzka, Martina V and Bernardes-Souza, Breno and Schlenk, Richard F and Schadendorf, Dirk	Journal of Medical Internet Research	A Face-Aging App for Smoking Cessation in a Waiting Room Setting: Pilot Study in an HIV Outpatient Clinic	Specialty Clinic	1	2018	DE	O	an intervention for smoking cessation that would make use of the time patients spend in a waiting room by passively exposing them to a face-aging, public morphing, tablet-based app, to pilot the intervention in a waiting room of an HIV outpatient clinic, and to measure the perceptions of this	Health Information	Smoking

Author	Journal	Title	Setting	# Kiosks	Yr	Country	Access	Comments	Purpose	Condition
								intervention among smoking and nonsmoking HIV patients. A face-aging app implemented in a waiting room provides a novel opportunity to motivate patients visiting a health care provider to quit smoking, to address quitting at their subsequent appointment and thereby encourage physician-delivered smoking cessation, or not to take up smoking.		
Chen, Milton	Telehealth and Medicine Today	How to Make Money in Telehealth	Community	NA	2018	US	O/R	How can enterprising businesses take advantage of telehealth without going broke? Within the next five years, we′ll see telehealth kiosks everywhere. Imagine talking to a doctor at your	Telehealth	Various

Author	Journal	Title	Setting	# Kiosks	Yr	Country	Access	Comments	Purpose	Condition
								convenience from an easy-to-use kiosk at work, in a grocery store, or a pharmacy. But HealthSpot, the pioneer in this space, failed despite raising around $50M of investment. HealthSpot used Vidyo, which is so complex and expensive that it broke the HealthSpot business model. Kiosks will need to be very inexpensive, less than $5,000 per kiosk.		
Elers, Phoebe	PhD Thesis	e-Healthcare: A critical examination of the patient portal initiative in New Zealand	Hospital	1	2018	NZ	R	Health kiosk Allows patients to self-register at a hospital and have their height, weight, BMI, blood pressure, pulse and oxygen saturation measured electronically and was introduced to Thames Hospital	Patient registration	Various

Author	Journal	Title	Setting	# Kiosks	Yr	Country	Access	Comments	Purpose	Condition
								in November 2014		
Hopfer, Suellen and Ray, Anne E and Hecht, Michael L and Miller-Day, Michelle and Belue, Rhonda and Zimet, Gregory and Evans, W Douglas and McKee, Francis X	Translational Behavioral Medicine	Taking an HPV vaccine research-tested intervention to scale in a clinical setting	Specialty Clinic	1	2018	US	O	Stakeholder engagement; developing narratives into prevention messages; kiosk design; usability of kiosk; waiting room observational study - kiosk was easily implemented, kiosk in sleep mode - ppl did not approach as thought it was switched off - kiosk was ignored in waiting room - the kiosk received little use even with receptionist prompting.	Health Information	Sexual Health
Kwok, Maria Y and Bakken, Suzanne R and Pusic, Martin V and York, Deborah and Pahalyants, Vartan and Ye, Choong and Javed, Kainaat and Dayan, Peter S	Pediatric Emergency Care	Development and Pilot Testing of a Computerized Asthma Kiosk to Initiate Chronic Asthma Care in a Pediatric Emergency Department	Emergency Department	1	2018	US	R	developed, itera-tively refined, and pilot tested the feasibility of a computerized asthma kiosk to (1) capture asthma information, (2) deliver asthma education, and	Health Information	Asthma

Author	Journal	Title	Setting	# Kiosks	Yr	Country	Access	Comments	Purpose	Condition
								(3) facilitate guideline-based chronic asthma management. A rigorously developed asthma kiosk showed promise for initiating chronic asthma care in the ED		
Meighan, Melissa M	Journal of Neuroscience Nursing	Stroke Education Video Does Not Affect Patient Satisfaction Scores	Hospital	2	2018	US	R	Patient satisfaction of educational materials - video +print vs print only. No significant differences between the two. Interaction with a nurse was important for some and didn't happen with the video	Health Information	Cardiovascular Disease
Mudumba, Rajeev	Telehealth and Medicine Today	HealthSpot: Analysis of a Bankruptcy in Kiosk-based Telehealth	Multiple	NA	2018	US	R	HealthSpot, Inc., the kiosk-based telehealth services provider founded in 2010. kiosks came equipped with proprietary cloud- based software, high-definition video conferencing, and interactive	Telehealth	Various

Author	Journal	Title	Setting	# Kiosks	Yr	Country	Access	Comments	Purpose	Condition
								medical devices. despite the capital it raised, and the name brand partners it brought together, HealthSpot closed up shop in December 2015. Reasons for failure: Did HealthSpot spend too long just proving their kiosks functionality in academic surroundings rather than vetting their business model in the market to bring in revenues early on? HealthSpot required its customers to have pre-scheduled appointments with physicians before availing themselves of services a HealthSpot's kiosk. HealthSpot had not yet planed for scaling.		

Author	Journal	Title	Setting	# Kiosks	Yr	Country	Access	Comments	Purpose	Condition
O'Neil, Owen and Fernandez, Manuel Murie and Herzog, J{\"{u}}rgen and Beorchia, Marta and Gower, Valerio and Gramatica, Furio and Starrost, Klaus and Kiwull, Lorenz	PM{\&}R	Virtual Reality for Neurorehabilitation: Insights From 3 European Clinics	Specialty Clinic	1	2018	DE	R	Observational study - some technological issues hindered use (e.g. sensors not working), some children weren’t tall enough for the VR, high motivation to use it, overall + perception	Motor rehabilitation	Pediatrics
Ullmann, Gerhild and Kedia, Satish K and Homayouni, Ramin and Akkus, Cem and Schmidt, Michael and Klesges, Lisa M and Ward, Kenneth D	BMC Public Health	Memphis FitKids: implementing a mobile-friendly web-based application to enhance parents' participation in improving child health	Community	5	2018	US	O	Implementation study: Kiosk has built in 'fitcheck' tool which collects weight, diet, physical activity, screen time and sleep habits. Produces tailored information including low-cost neighborhood resources to support healthy lifestyle. Uses GIS. 38,429 fitcheck sessions were completed between 2014-2016. Web based health tools inc. kiosks viable way to increase access to info about health	Clinical Measurements	Pediatrics

Author	Journal	Title	Setting	# Kiosks	Yr	Country	Access	Comments	Purpose	Condition
								weight and lifestyle in children		
Valdez, Armando and Napoles, Anna M and Stewart, Susan L and Garza, Alvaro	Journal of Cancer Education	A Randomized Controlled Trial of a Cervical Cancer Education Intervention for Latinas Delivered Through Interactive, Multimedia Kiosks	Primary Care	3	2018	US	R	study employed a randomized controlled trial design to test the efficacy of an interactive multimedia cervical cancer education intervention compared to a usual care control group. Study suggests that interactive touchscreen kiosks are an effective cancer education medium	Health Information	Cervical Cancer
Antonio, Marcy G and Courtney, Karen L and Lingler, Jennifer H and Matthews, Judith T	Studies in health technology and informatics	Translating Behavior Change Techniques to New Delivery Mediums.	Senior Center	1	2017	US, CA	R	The Community Multi-User Health Kiosk was initially designed to assist older community-dwelling adults in monitoring their health and communicating with their health care providers. The communication module within a	Health Information	Various

Author	Journal	Title	Setting	# Kiosks	Yr	Country	Access	Comments	Purpose	Condition
								multi-user telehealth kiosk will be used as an example of how behavior change techniques from a "live" intervention may be represented in an information technology-delivered intervention.		
Bahadin, Juliana and Shum, Eugene and Ng, Grace and Tan, Nicolette and Sellayah, Pushpavalli and Tan, Sze Wee	Journal of General Internal Medicine	Follow-Up Consultation Through a Healthcare Kiosk for Patients with Stable Chronic Disease in a Primary Care Setting: A Prospective Study	Primary Care	1	2017	SG	R	participants used the kiosk instead of consulting a physician. All participants who used the kiosk were also evaluated by a nurse clinician (NC). Healthcare kiosks can potentially be used to complement primary care clinician visits for man- aging patients with stable chronic diseases	Clinical Measurements	Various
Boos, Johannes and Fang, Jieming and Snell, Aideen and Hallett, Donna and Siewert, Bettina and	American Journal of Roentgenology	Electronic Kiosks for Patient Satisfaction Survey in Radiology	Hospital	1	2017	US	R	analyze patient satisfaction surveys obtained via electronic kiosks in a	Survey Administration	Radiology

Author	Journal	Title	Setting	# Kiosks	Yr	Country	Access	Comments	Purpose	Condition
Eisenberg, Roland L and Brook, Olga R								tertiary-care academic radiology department. Survey kiosks led to a higher response rate than online surveys. The completion rate can be further improved by placing kiosks next to elevators.		
Frail, Caitlin K and Cooper, Susan and Gallagher, Tim and Sarkis, Josh and Topor, Laura and Bruzek, Richard J	Journal of the American Pharmacists Association	A technology-supported collaboration between a health plan and a community pharmacy to improve blood pressure control	Pharmacy	9	2017	US	R	Pilot study. Results demonstrated promising early results in a model that has potential to improve blood pressure monitoring and management in community setting	Clinical Measurements	Hypertension
Gleason-Comstock, Julie and Streater, Alicia and Goodman, Allen and Janisse, James and Brody, Aaron and Mango, LynnMarie and Dawood, Rachelle and Levy, Phillip	BMC Health Services Research	Willingness to pay and willingness to accept in a patient-centered blood pressure control study	Emergency Department	1	2017	US	R	RCT was to determine if enhanced discharge from the emergency department using kiosk-based hypertension education modules would improve patient blood pressure	Clinical Measurements	Hypertension

Author	Journal	Title	Setting	# Kiosks	Yr	Country	Access	Comments	Purpose	Condition
								control. This approach utilizing WTP and WTA could be of value.		
Hopfer, Suellen and Hecht, Michael and Ray, Anne and Miller-Day, Michelle and BeLue, Rhonda and Zimet, Greg	IMPLEMENTATION SCIENCE	Feasibility of implementing a community clinic based interactive health kiosk about HPV vaccination targeting African American young adult women attending Planned Parenthood	Specialty Clinic	1	2017	US	O	narrative video intervention embedded within an interactive health kiosk to be used for waiting or exam rooms that aimed to increase HPV vaccine uptake. results from the observational study suggested placement in the waiting room would not result in use of the intervention.	Health Information	Sexual Health
Hsieh, Yu-Hsiang and Beck, Kaylin J and Rothman, Richard E and Gauvey-Kern, Megan and Woodfield, Alonzo and Peterson, Stephen and Signer, Danielle and Gaydos, Charlotte A	International Journal of STD {\&} AIDS	Factors associated with patients who prefer HIV self-testing over health professional testing in an emergency department-based rapid HIV screening program	Emergency Department	1	2017	US	R	Kiosk-facilitated HIV self-testing has been shown to be accurate and well accepted by emergency department (ED) patients. We investigated factors associated with patients who preferred self-testing over testing	Screening	HIV

Author	Journal	Title	Setting	# Kiosks	Yr	Country	Access	Comments	Purpose	Condition
								performed by health professionals in an ED-based HIV screening program. 48% of patients chose to use the kiosk to guide them in self-testing. HIV self-testing in the ED could serve as a complementary testing approach to the conventional modality.		
Lewis, Mitra K and Hsieh, Yu-Hsiang and Gaydos, Charlotte A and Peterson, Stephen C and Rothman, Richard E	International Journal of STD {\&} AIDS	Informed consent for opt-in HIV testing via tablet kiosk: an assessment of patient comprehension and acceptability	Emergency Department	1	2017	US	R	cross-sectional survey study examined patient comprehension of opt-in HIV testing consent and acceptability of using a kiosk to provide consent Subjects reported they were comfortable using the kiosk, found the kiosk easy to use, and reported a positive experience using the kiosk.	Patient registration	HIV
Lopez, Gabriela G	Doctoral	Integrating	Community	NA	2017	US	R	explore the	Clinical	Health and

Author	Journal	Title	Setting	# Kiosks	Yr	Country	Access	Comments	Purpose	Condition
Andrade	Dissertation	Technology to Improve Health and Well-Being in the Latino Population						utilization of the IDEAL LIFE Interactive Kiosk� to improve the health and well-being of enrolled participants. A quasi-experimental, pre-test and post-test design was utilized. this study has suggested that technology is playing a big role in assisting with self-monitoring. The research points to the potential value of technology to improve self-monitoring, health, and well-being. Further	Measurements	Wellbeing
Mackrill, J and Marshall, P and Payne, S R and Dimitrokali, E and Cain, R	Applied ergonomics	Using a bespoke situated digital kiosk to encourage user participation in healthcare environment design.	Hospital	1	2017	UK	O	Kiosk to gather user participation in eliciting ideas for improving the health care environment. Kiosk was successful in providing a platform for interacting with	Participatory design	Healthcare environment

Author	Journal	Title	Setting	# Kiosks	Yr	Country	Access	Comments	Purpose	Condition
								users to design the health care environment		
Schwebel, David C and Severson, Joan and He, Yefei	Virtual Reality	Using smartphone technology to deliver a virtual pedestrian environment: usability and validation	Laboratory	1	2017	US	R	Observational comparative study of Semi virtual reality kiosk vs smartphone VR to teach pedestrian safety Convergent validity was detected, with many aspects of pedestrian behavior in the smartphone-based virtual environment matching behavior in the kiosk virtual environment.	Health Information	Pediatric Injuries
Silva, Joao and Brandao, Pedro and Prior, Rui	2017 IEEE Symposium on Computers and Communications (ISCC)	Usability assessment of a Health Kiosk	Multiple	NA	2017	PT	O/R	developed a modular and reconfigurable Health Kiosk based on such devices, where users can autonomously measure vital signs for screening or continued monitoring. present an assessment of its	Clinical Measurements	Various

Author	Journal	Title	Setting	# Kiosks	Yr	Country	Access	Comments	Purpose	Condition
								usability testing into four parts: (a) clicks performed and their location, (b) time taken using the kiosk, (c) observation report, and (d) final question-naire. The global acceptance of the system by the participants was very positive, with only a few aspects in need of improvement.		
Sousa, P and Rodrigues, J and Brand{\~{a}}o, P	NA	62. HEALTH KIOSK: What factors influence the decision on how and when to use it?	Primary Care	1	2017	PT	R	The study aimed to identify factors that influence the decision on how and when to use a "Health Kiosk". The target population were users of the primary healthcare facility. Citizens who tried the kiosk mostly found it useful, easy to use, credible and secure.	Clinical Measurements	Various
Takyi, Harold and Watzlaf, Valerie and	International journal of	Privacy and Security in Multi-User Health	Community	NA	2017	US	O/R	for creating and maintaining a	Health Information,	Various

Author	Journal	Title	Setting	# Kiosks	Yr	Country	Access	Comments	Purpose	Condition
Matthews, Judith Tabolt and Zhou, Leming and Dealmeida, Dilhari	telerehabilitation	Kiosks.						security and privacy audit checklist for multi-user health kiosks. Implementation of selected audit elements for a multi-user health kiosk designed for use by community-residing older adults illustrates how the guide can be applied.	Clinical measurements	
Chung, Chia Fang and Munson, Sean A. and Thompson, Matthew J. and Baldwin, Laura Mae and Kaplan, Jeffrey and Cline, Randall and Green, Beverly B.	Journal of the American Board of Family Medicine	Implementation of a new kiosk technology for blood pressure management in a family medicine clinic: From the WWAMI region practice and research network	Primary Care	2	2016	US	R	objective of this study was to evaluate BP kiosk acceptability and usability, as well as its effects on the workflow of patient BP self-measurement in a primary care clinic. Providers, staff, and patients adapted to the use of BP kiosks, providing value by keeping patients in their own care and saving MA time.	Clinical Measurements	Hypertension
Hossain, Rosa and Wilson, Colin and Hatzidimitriadou,	NA	Report on the �Health MOT Roadshow'Communi	Community	1?	2016	UK	O/ R	NHS community health checks, Health MOT's	Clinical Measurements	Cardiovascular Disease

Author	Journal	Title	Setting	# Kiosks	Yr	Country	Access	Comments	Purpose	Condition
Eleni		ty Health Check Project						The �Health MOT Roadshow� is a community-based health check program consisting of outreach, referrals, and on-site Health MOTs as well as NHS Health Check.		
Hsieh, Yu-Hsiang and Holtgrave, David R and Peterson, Stephen and Gaydos, Charlotte A and Rothman, Richard E	AIDS Care	Novel emergency department registration kiosk for HIV screening is cost-effective	Emergency Department	1	2016	US	R	A two-phase quasi experimental design. Phase 1 and 2 lasted 2 months after which they calculated estimated cost (cost-effective ratio- CER)	Screening	HIV
Joshi A, Amadi C, Meza J, Aguire T	Int J Med Inform	Evaluation of a computer-based bilingual breastfeeding educational program on breastfeeding knowledge, self-efficacy and intent to breastfeed among rural Hispanic women	Specialty Clinic	1	2016	US	R	Hispanic women living in rural settings showed improvement in breastfeeding knowledge, self-efficacy and intent to breastfeed using the computer based bi-lingual educational program.	Health Information	Breastfeeding
Lee, Joo-Young and Jung, Dooyoung and	Psycho-Oncology	Correlates of oncologist-issued	Specialty Clinic	1	2016	KR	O	Using touch-screen kiosks at	Screening	Mental Health

Author	Journal	Title	Setting	# Kiosks	Yr	Country	Access	Comments	Purpose	Condition
Kim, Won-Hyoung and Lee, Hyuk-Joon and Noh, Dong-Young and Hahm, Bong-Jin		referrals for psycho-oncology services: what we learned from the electronic voluntary screening and referral system for depression (eVSRS-D)						a tertiary hospital in Korea, participants with cancer completed the Patient Health Questionnaire-9 at their convenience, received the results, and reported their willingness to participate in POS. The eVSRS-D cannot definitively diagnose major depression but may efficiently self- select a population with significant depression symptoms.		
Ng, Grace and Tan, Nicolette and Bahadin, Juliana and Shum, Eugene and Tan, Sze Wee	Journal of Medical Systems	Development of an Automated Healthcare Kiosk for the Management of Chronic Disease Patients in the Primary Care Setting	Primary Care	1	2016	SG	R	One-hundred patients with stable chronic disease were recruited from a primary care clinic. They used a kiosk in place of doctors� consultations for two subsequent follow-up visits. Healthcare kiosks represent	Clinical Measurements	Various

Author	Journal	Title	Setting	# Kiosks	Yr	Country	Access	Comments	Purpose	Condition
								an alternative way to manage patients with stable chronic disease. They have the potential to replace physician visits and improve access to primary healthcare.		
Nicholas, D and Huntington, P and Williams, P	NA	Digital health information for the consumer: evidence and policy implications	Review	NA	2016	UK	O/R	Review of Health Kiosk implementations in the UK. The role of kiosks in providing health information was limited by a relatively poor take-up amongst the general public and the limited static menu and content. Kiosks were also associated with limited health outcomes. Internet users did use the platform for health information.	Health Information	Various
Orlando, Megan S and Rothman, Richard E and Woodfield, Alonzo and Gauvey-	The Journal of Emergency Medicine	Public Health Information Delivery in the Emergency Department:	Emergency Department	1	2016	US	R	The kiosk module contained five screens: 1)	Health Information	Various

Author	Journal	Title	Setting	# Kiosks	Yr	Country	Access	Comments	Purpose	Condition
Kern, Megan and Peterson, Stephen and Miller, Tammi and Hill, Peter M and Gaydos, Charlotte A and Hsieh, Yu-Hsiang		Analysis of a Kiosk-Based Program						manual login screen; 2) survey of patients� interest (yes/no) in receiving information about health topics; 3) interest in updating health information via kiosk; 4) HIV test offer; and 5) relevant instructions dependent on acceptance of HIV test.		
Shields, Wendy C and McDonald, Eileen M and McKenzie, Lara B and Gielen, Andrea C	Clinical Pediatrics	Does Health Literacy Level Influence the Effectiveness of a Kiosk-Based Intervention Delivered in the Pediatric Emergency Department?	Emergency Department	1	2016	US	R	a randomized trial of an injury prevention intervention delivered via computer kiosk in a pediatric emergency department. Participants reading level had an independent and significant effect on safety knowledge outcomes.	Health Information	Pediatric Injuries
Soares, Eduardo and Oliveira, Cristina and Maia, Joao and Almeida, Rafael and Coimbra, Miguel and	2016 IEEE Symposium on Computers and Communicatio n (ISCC)	Modular Health Kiosk for health self-assessment	Community	4	2016	BR. PT	O	building a Health Kiosk from commercial, off-the shelf Personal Health	Clinical Measurements	Various

Author	Journal	Title	Setting	# Kiosks	Yr	Country	Access	Comments	Purpose	Condition
Brandao, Pedro and Prior, Rui								Devices and a computer with a touch- screen interface. The prototype was tested in multiple events with different user groups		
Thomas, Alex	NA	Access and Use: Improving Digital Multimedia Consumer Health Information.	Community	1	2016	AU	O	Measurement of the actual use of consumer health information (CHI) content through Kaplan-Meir survival plots. Clear patterns of abandonment of content use were exhibited.	Health Information	Various
Venkatesh, Viswanath and Rai, Arun and Sykes, Tracy Ann and Aljafari, Ruba	MIS Quarterly	Combating Infant Mortality in Rural India: Evidence from a Field Study of eHealth Kiosk Implementations	Community	10	2016	IN	O	Examines how the use of one ICT intervention specifically, eHealth kiosks disseminating authenticated and accessible medical information can alleviate the problem of high infant mortality in rural India. The results revealed that (1) eHealth kiosk use promotes	Health Information	Infant Mortality

Author	Journal	Title	Setting	# Kiosks	Yr	Country	Access	Comments	Purpose	Condition
								seeking professional medical care and reduces infant mortality,		
Belt, Katrina	Health management technology	How self-service check-in works in the real world. Baptist Health CFO Katrina Belt gives the in- sider scoop on kiosk adoption and use by staff and patients.	Specialty Clinic	8	2015	US	R	Patient self-check-in kiosks have reduced check-in time to two minutes on the average.	Patient registration	Various
Courtney, Karen L and Matthews, Judith T and McMillan, Julie M and {Person Mecca}, Laurel and Smailagic, Asim and Siewiorek, Daniel	Studies in health technology and informatics	Usability testing of a prototype multi-user telehealth kiosk.	Retirement community	1	2015	US	O	Usability testing on a prototype telehealth kiosk	Telehealth	Various
Hankin, Abigail and Haley, Leon and Baugher, Amy and Colbert, Kia and Houry, Debra	Western Journal of Emergency Medicine	Kiosk versus In-person Screening for Alcohol and Drug Use in the Emergency Department: Patient Preferences and Disclosure	Emergency Department	1	2015	US	O	ED patients were significantly more likely to disclose at-risk alcohol and substance use to a computer kiosk than an interviewer.	Screening	Alcohol and drug use
Joshi, Ashish and Amadi, Chioma and Meza, Jane and Aguirre, Trina and Wilhelm, Sue	Journal of Community Health	Comparison of Socio-Demographic Characteristics of a Computer Based Breastfeeding Educational Intervention Among Rural Hispanic Women	Specialty Clinic	1	2015	US	R	Participants (n=23) received bi-lingual breastfeeding education using touch screen computer and printed educational	Health Information	Breastfeed ing

Author	Journal	Title	Setting	# Kiosks	Yr	Country	Access	Comments	Purpose	Condition
								material.		
Joshi, Ashish; Perin, Douglas M. Puricelli; Amadi, Chioma; Trout, Kate	Journal of innovation in health informatics	Evaluating the usability of an interactive, bi-lingual, touchscreen-enabled breastfeeding educational programme: application of Nielson's heuristics.	Specialty Clinic	1	2015	US	R	Kiosk System for Breastfeeding Education	Health Information	Breastfeeding
Lesselroth, Blake and Adams, Kathleen and Tallett, Stephanie and Ragland, Scott and Church, Victoria and Borycki, Elizabeth M and Kushniruk, Andre	NA	Usability Evaluation of a Medication Reconciliation and Allergy Review (MRAR) Kiosk: A Methodological Approach for Analyzing User Interactions	Specialty Clinic	1	2015	US	R	Assessing usability of a patient-facing medication reconciliation and allergy review (MRAR) kiosk. presented the framework for a mixed-method assessment combining heuristic evaluation with �typical� usability testing.	Medication reconciliation	Medication
Padwal, Raj S and Townsend, Raymond R and Trudeau, Luc and Hamilton, Peter G and Gelfer, Mark	Journal of the American Society of Hypertension	Comparison of an in-pharmacy automated blood pressure kiosk to daytime ambulatory blood pressure in hypertensive subjects	Pharmacy	1	2015	CA	R	The PharmaSmart PS�2000 closely approximated mean daytime ambulatory BP, supporting the use of serial readings from this device in the	Clinical Measurements	Hypertension

Author	Journal	Title	Setting	# Kiosks	Yr	Country	Access	Comments	Purpose	Condition
								assessment of BP		
Salim, Ali and Berry, Cherisse and Ley, Eric J and Schulman, Danielle and Anderson, Jacqueline and Navarro, Sonia and Zheng, Ling and Chan, Linda S	Health Education Journal	Improving organ donor registration using kiosks in primary care clinics	Primary Care	4	2015	US	O	Kiosks containing organ donation educational material were set up at each clinic for a total of 7 weeks. compared with unstaffed kiosks, staffed kiosks are more effective in increasing organ donation registration	Patient registration	Organ donation
Schluter, Philip and Lee, Martin and Hamilton, Greg and Coe, Gill and Messer-Perkins, Heather and Smith, Belinda	Journal of Public Health Dentistry	Keep on brushing: a longitudinal study of motivational text messaging in young adults aged 18-24 years receiving Work and Income Support	Community	1	2015	NZ	O	unemployed young adults aged 18�24 years with access to a mobile phone were recruited using either a purpose-built computer kiosk or Work and Incomes Facebook site. Participants	Health Information	Dental Health
Serafico, Michael E	NA	114: UTILIZATION OF AN AUTOMATED HEALTH KIOSK IN ESTIMATING THE INCIDENCE OF OVERWEIGHT AND OBESITY: AN ALTERNATIVE	Pharmacy	4	2015	PH	O	The automated health kiosk can be used an alternative device to estimate the incidence of overweight and obesity in the	Clinical Measurements	Obesity

Author	Journal	Title	Setting	# Kiosks	Yr	Country	Access	Comments	Purpose	Condition
		TECHNIQUE FOR COMMUNITY-BASED HEALTH-RISK ASSESSMENT						community.		
Trick, W. E.; Deamant, C.; Smith, J.; Garcia, D.; Angulo, F.	Applied clinical informatics	NA	Specialty Clinic	NA	2015	US	R	Kiosk for collecting patient outcome measures in general medicine clinic	Patient Outcomes Data	General Medicine
Wrenn, Glenda and Kasiah, Fatima and Syed, Irshad	Journal of innovation in health informatics	Using a self-service kiosk to identify behavioural health needs in a primary care clinic serving an urban, underserved population.	Primary Care	1	2015	US	R	feasibility of using a kiosk placed in a primary care clinic to screen for multiple mental health disorders.	Screening	Mental Health
Youm, Sekyoung and Park, Seung-Hun	Telemedicine journal and e-health : the official journal of the American Telemedicine Association	How the awareness of u-healthcare service and health conditions affect healthy lifestyle: an empirical analysis based on a u-healthcare service experience.	Community	1	2015	KR	O	u-healthcare center, a kiosk, devices for health checkup, a body-type examination system, and a physical fitness assessment system were installed. To pro- mote the usage of u-healthcare service, the understanding of the service and the credibility of examination results need to be in- creased by	Clinical Measurements	Health and Wellbeing

Author	Journal	Title	Setting	# Kiosks	Yr	Country	Access	Comments	Purpose	Condition
								sharing successful cases. Furthermore,		
{Yvonne Chan}, Yu-Feng and Nagurka, Roxanne and Bentley, Suzanne and Ordonez, Edgardo and Sproule, William	Health promotion perspectives	Medical utilization of kiosks in the delivery of patient education: a systematic review.	Review	NA	2014	Various	O/R	Touch screen kiosks are effective in educating patients and in improving healthcare, both at a performance and cost advantage over other modes of patient education.	Health Information	Various
Ahn, Ho Seok and Kuo, I-Han and Datta, Chandan and Stafford, Rebecca and Kerse, Ngaire and Peri, Kathy and Broadbent, Elizabeth and MacDonald, Bruce A	SIMULATION, MODELING, AND PROGRAMMING FOR AUTONOMOUS ROBOTS (SIMPAR 2014)	Design of a Kiosk Type Healthcare Robot System for Older People in Private and Public Places	Retirement community	1	2014	NZ	O	The kiosk type service robot platform is used for giving helpful information to older people through a touch screen.	Telehealth	Various
Alpert, Bruce S and Dart, Richard A and Sica, Domenic A	Journal of the American Society of Hypertension	Public-use blood pressure measurement: the kiosk quandary	Community	NA	2014	US	O/R	Two criterial for a clinically acceptable BP Kiosk are: proper validation testing to an accepted national standard, and a cuff that is suitable for the particular arm circumference of the patient.	Clinical Measurements	Hypertension

Author	Journal	Title	Setting	# Kiosks	Yr	Country	Access	Comments	Purpose	Condition
Brixey, Suzanne N and Weaver, Nancy L and Guse, Clare E and Zimmermann, Haydee and Williams, Janice and Corden, Timothy E and Gorelick, Marc H	Clinical Pediatrics	The Impact of Behavioral Risk Assessments and Tailored Health Information on Pediatric Injury	Primary Care	1	2014	US	R	Kiosk administered injury prevention self-assessment tool successfully integrated into a pediatric practice.	Health Information	Pediatric Injuries
Dulchavsky, Scott A and Ruffin, Wilma J and Johnson, Dayna A and Cogan, Chad and Joseph, Christine L M	Frontiers in Public Health	Use of an Interactive, Faith-Based Kiosk by Congregants of Four Predominantly, African\^a��American Churches in a Metropolitan Area	Community	4	2014	US	O	Congregants were surveyed to describe kiosk-use, kiosk-user characteristics, health status, and self-reported behavior changes attributed to the kiosk. Knowledge of kiosk-user characteristics and the �health status� of a congregation, provide an opportunity for targeted, church-based health promotion	Health Information	Various
Gittelman, Michael A and Pomerantz, Wendy J and McClanahan, Nicole and Damon, Alison and Ho, Mona	Journal of Trauma and Acute Care Surgery	A computerized kiosk to teach injury prevention	Emergency Department	1	2014	US	R	Kiosk for screening injury risk. Behavior changes are significantly more if seen by injury specialist than by kiosk alone.	Health Information	Pediatric Injuries
Greiner, K. Allen;	American	Implementation	Primary	9	2014	US	R	Kiosk to educate	Health	Cancer

Author	Journal	Title	Setting	# Kiosks	Yr	Country	Access	Comments	Purpose	Condition
Daley, Christine M.; Epp, Aaron; James, Aimee; Yeh, Hung-Wen; Geana, Mugur; Born, Wendi; Engelman, Kimberly K.; Shellhorn, Jeremy; Hester, Christina M.; LeMaster, Joseph; Buckles, Daniel C.; Ellerbeck, Edward F.	journal of preventive medicine	intentions and colorectal screening: a randomized trial in safety-net clinics.	Care					patients about colorectal cancer screening	Information	
Hsieh, Yu-Hsiang and Gauvey-Kern, Megan and Peterson, Stephen and Woodfield, Alonzo and Deruggiero, Katherine and Gaydos, Charlotte A and Rothman, Richard E	Journal of telemedicine and telecare	An emergency department registration kiosk can increase HIV screening in high risk patients.	Emergency Department	1	2014	US	R	ED-based HIV screening via a registration-based kiosk was feasible, yielded similar proportions of testing, and increased the proportion of engagement of higher-risk patients in testing.	Screening	HIV
Joshi, Ashish and Trout, Kate	Health Information {\&} Libraries Journal	The role of health information kiosks in diverse settings: a systematic review	Review	NA	2014	Various	O/R	31 Articles included in review. Majority were in urban settings, clinical settings and focused on child safety. The results suggest that health information kiosks are a feasible medium to disseminate	Health Information	Various

Author	Journal	Title	Setting	# Kiosks	Yr	Country	Access	Comments	Purpose	Condition
								health information		
Langley, Christopher A and Bush, Joseph and Patel, Alpa	NA	An evaluation: the implementation and impact of healthy living pharmacies within the Heart of Birmingham	Pharmacy	10	2014	UK	O	a service-user questionnaire deployed via touchscreen kiosks located within the ten	Health Information	Health and Wellbeing
Mahnke, Andrea N and Plasek, Joseph M and Hoffman, David G and Partridge, Nathan S and Foth, Wendy S and Waudby, Carol J and Rasmussen, Luke V and McManus, Valerie D and McCarty, Catherine A	American Journal of Medical Genetics Part A	A rural community's involvement in the design and usability testing of a computer-based informed consent process for the personalized medicine research project	Specialty Clinic	1	2014	US	R	Obtaining informed consent via computer kiosk for a population based genetic study. A computer-based consent may serve to better communicate consistent, clear, accurate, and complete information.	Informed Consent	Genetic Study
Ong, Stephanie and Min, Kelly and Porter, Eveline and Jassal, Vanita and Logan, Alexander and Miller, Judith	American Journal of Kidney Diseases	QUALITATIVE EVALUATION OF A PATIENT SELF-MANAGEMENT KIOSK USE IN ADVANCED CHRONIC KIDNEY DISEASE (CKD) FOR A 3-YEAR PERIOD	Specialty Clinic	1	2014	CA	R	Results show that Self-management kiosk for chronic kidney disease added quality to the visit.	Disease monitoring	Chronic Kidney Disease
Orlando, Megan S and Rothman, Richard E and Woodfield, Alonzo and Gauvey-Kern, Megan and Peterson, Stephen and Hill, Peter M and	The American journal of emergency medicine	Kiosks as tools for health information sharing: exploratory analysis of a novel ED program.	Emergency Department	1	2014	US	R	findings suggest promise for use of kiosks in the ED as a supportive communication tool with the	Screening	HIV

Author	Journal	Title	Setting	# Kiosks	Yr	Country	Access	Comments	Purpose	Condition
Gaydos, Charlotte A and Hsieh, Yu-Hsiang								majority of patients expressing comfort with using the kiosk to share health information with providers. Self-service kiosks have the potential to speed up ED visits because patients can fill in medical and surgical history and medication lists during wait times.		
Pack, Jeri	Health management technology	Using self-service kiosks with your check-in staff. The right combination for higher patient care.	Specialty Clinic	Unknown	2014	US	R	Hassles have been eliminated by implementing several self-registration kiosks. kiosks do not replace staff. They enhance what they can do.	Patient registration	Cancer
Rosas, Lisa G. and Trujillo, Celina and Camacho, Jose and Madrigal, Daniel and Bradman, Asa and Eskenazi, Brenda	Patient Education and Counseling	Acceptability of health information technology aimed at environmental health education in a prenatal clinic	Specialty Clinic	1	2014	US	R	An interactive computer kiosk that provides environmental health education to low-income Latina prenatal patients was found to be acceptable.	Health Information	Environmental health

Author	Journal	Title	Setting	# Kiosks	Yr	Country	Access	Comments	Purpose	Condition
Rothman, Richard E and Gauvey-Kern, Megan and Woodfield, Alonzo and Peterson, Stephen and Tizenberg, Boris and Kennedy, Joseph and Bush, Devon and Locke, William and Gaydos, Charlotte A and Deruggiero, Katherine and Hsieh, Yu-Hsiang	Telemedicine and e-Health	Streamlining HIV Testing in the Emergency Department�Leveraging Kiosks to Provide True Universal Screening: A Usability Study	Emergency Department	1	2014	US	R	This study evaluated the usability of kiosks within the existing HIV testing program and assessed patients� perceived acceptability of kiosk-based screening in the ED. Response was favorable.	Screening	HIV
Shafii, Taraneh and Benson, Samantha K and Morrison, Diane M and Hughes, James P and Golden, Matthew R and Holmes, King K	Journal of Adolescent Health	Results from eKISS (electronic KIOSK Intervention for Safer-Sex): A Pilot Randomized Controlled Trial to Test an Interactive Computer-Based Intervention for Sexual Health in Adolescents and Young Adults	Specialty Clinic	1	2014	US	R	Kiosk based intervention for sexual health. Kiosk users reported greater practice of safe sex 3 months post intervention than control group.	Health Information	Sexual Health
Sinha, Madhumita and Khor, Kai-Ning and Amresh, Ashish and Drachman, David and Frechette, Alan	Pediatric emergency care	The use of a kiosk-model bilingual self-triage system in the pediatric emergency department.	Emergency Department	1	2014	US	R	Kiosk triage enabled users to enter basic medical triage history data quickly and accurately in an ED setting with	Patient Triage	Pediatrics
Tse, Julia and Nansel, Tonja R and Weaver, Nancy L and Williams, Janice and Botello-Harbaum,	Health promotion practice	Implementation of a tailored kiosk-based injury prevention program in pediatric primary care.	Specialty Clinic	5	2014	US	R	Qualitative study to identify barriers and facilitators to a kiosk based child	Health Information	Pediatric Injuries

Author	Journal	Title	Setting	# Kiosks	Yr	Country	Access	Comments	Purpose	Condition
Maria								injury prevention program.		
{Al Hamarneh}, Yazid N and Houle, Sherilyn K.D. and Chatterley, Patricia and Tsuyuki, Ross T	Blood Pressure Monitoring	The validity of blood pressure kiosk validation studies	Review	NA	2013	Various	O/R	Systematic review of nine studies about blood pressure kiosks. Only one reported adherence validation standards.	Review	Hypertension
Ahlers-Schmidt, Carolyn R and Jones, Jordan T and Chesser, Amy and Weeks, Kerri	Telemedicine and e-Health	Evaluating Opportunities for Text Message Communication: A Survey of Parents and Teens	Specialty Clinic	1	2013	US	R	Survey on acceptability of text message communication for teens and parents	Survey Administration	Patient communication
Bean, Kristen and Davis, Olga and Valdez, Hector	The Journal of Community Informatics	Bridging the Digital Divide: A Bilingual Interactive Health Kiosk for Communities Affected by Health Disparities	Community	2	2013	US	O	Bilingual kiosk giving information on HIV, mental health and substance abuse; FAQ page for posting questions, needs assessment survey,	Health Information	Various
Bolin, Jane N and Ory, Marcia G and Wilson, Ashley D and Salge, Lesley	The Diabetes Educator	Diabetes Education Kiosks in a Latino Community	Multiple	5	2013	US	O	Kiosk to deliver diabetes education at 5 sites. 3 out of 5 chose to continue using kiosks after end of study period	Health Information	Diabetes
Brys, Shannon	Behavioral healthcare	Kiosk-based "office" extends reach of health services	Multiple	NA	2013	US	O/R	News Item about Health Spot and CSI telehealth	Telehealth	Various

Author	Journal	Title	Setting	# Kiosks	Yr	Country	Access	Comments	Purpose	Condition
		providers.						kiosks		
Cohen, Amy N and Chinman, Matthew J and Hamilton, Alison B and Whelan, Fiona and Young, Alexander S	Medical Care	Using Patient-facing Kiosks to Support Quality Improvement at Mental Health Clinics	Specialty Clinic	4	2013	US	R	Routine data collection via kiosks for patients attending mental health clinics. Use of kiosks led to participants likely to use weight services.	Routine Data Collection	Mental Health
Demiris, George and Thompson, Hilaire and Boquet, Jaime and Le, Thai and Chaudhuri, Shomir and Chung, Jane	Informatics for Health and Social Care	Older adults' acceptance of a community-based telehealth wellness system	Retirement community	1	2013	US	R	Qualitative study on acceptance of telehealth kiosk in retirement community. Attitudes towards health monitoring were favorable.	Telehealth	Various
Demiris, George and Thompson, Hilaire J and Reeder, Blaine and Wilamowska, Katarzyna and Zaslavsky, Oleg	International journal of medical informatics	Using informatics to capture older adults' wellness.	Retirement community	1	2013	US	R	older adults are willing to participate in technology-enhanced interventions	Telehealth	Various
Doering, Tracey and Harwell, Susan and Fassler, Cheryl and Burr, Kesley and Hewitt, Sara and Trabue, Christopher	Journal of community hospital internal medicine perspectives	An interventional pilot study on obesity among low-income patients using a computer-based weight management module.	Primary Care	1	2013	US	R	Computerized educational module on weight management. Post-test scores improved but BMI was unaffected.	Health Information	Obesity
Gaydos, Charlotte A and Solis, Melissa and Hsieh, Yu-Hsiang and Jett-Goheen, Mary	International Journal of STD {\&} AIDS	Use of tablet-based kiosks in the emergency department to guide	Emergency Department	1	2013	US	R	Tablet-based kiosk for guiding patients in self-testing for HIV.	Screening	HIV

Author	Journal	Title	Setting	# Kiosks	Yr	Country	Access	Comments	Purpose	Condition
and Nour, Samah and Rothman, Richard E		patient HIV self-testing with a point-of-care oral fluid test						Tablet based tasting was feasible acceptable and accurate.		
Gleason-Comstock, Julie A and Streater, Alicia and Jen, Kai-Lin Catherine and Artinian, Nancy T and Timmins, Jessica and Baker, Suzanne and Joshua, Bosede and Paranjpe, Aniruddha	Patient Education and Counseling	Consumer health information technology in an adult public health primary care clinic: A heart health education feasibility study	Primary Care	1	2013	US	R	Kiosk for self-monitoring and education on heart health. Improvement was seen in weight and BP.	Health Information	Cardiovascular Disease
Joshi, Ashish and {Puricelli Perin}, Douglas M and Arora, Mohit	Rural and remote health	Using Portable Health Information Kiosk to assess chronic disease burden in remote settings.	Community	3	2013	IN	R	Chronic disease health risk assessment via kiosk in three types of settings. Subjective data and clinical measurements (weight, blood sugar and BP) taken. Kiosks can be used to assess chronic disease burden in the community.	Clinical Measurements	Various
McMillan, Julie M. and Courtney, Karen L. and Matthews, Judith T. and Smailagic, Asim and Siewiorek, Daniel	Studies in health technology and informatics	Designing the community multi-user health kiosk.	Community	1	2013	US	R	Multi-user kiosk for collecting clinical measurements and mental health assessments	Clinical Measurements	Various
Ong, Stephanie W. and Jassal, Sarbjit V. and Porter, Eveline	Seminars in Dialysis	Using an electronic self-management tool to support	Specialty Clinic	1	2013	CA	R	Kiosk for self-monitoring of chronic kidney	Disease monitoring	Chronic Kidney Disease

Author	Journal	Title	Setting	# Kiosks	Yr	Country	Access	Comments	Purpose	Condition
and Logan, Alexander G. and Miller, Judith A.		patients with chronic kidney disease (CKD): A CKD clinic self-care model						disease. Patients felt the kiosk positively impacted their clinic visit		
Raj, Tony and Sarah, Tinku and D, Dhinagaran and Ugargol, Allen P	Journal of Health Informatics in Developing Countries	Design, Development and Implementation of a Touch-Screen Health Information Kiosk for Patients at the Outpatient Waiting Area in a Large Tertiary Care Hospital in India: An Evaluation of User Satisfaction	Specialty Clinic	1	2013	IN	O	Health Information Kiosk. Ease of use, easy navigation, availability of information sought and the adequacy of health information available were found to be important parameters for user satisfaction	Health Information	Various
Sano, Mary and Egelko, Susan and Donohue, Michael and Ferris, Steven and Kaye, Jeffrey and Hayes, Tamara L and Mundt, James C and Sun, Chung-Kai and Paparello, Silvia and Aisen, Paul S	Alzheimer Disease {\&} Associated Disorders	Developing Dementia Prevention Trials	Home	178	2013	US	R	Dementia assessment via kiosk installed in home (one of three treatment arms). Higher dropout rate in kiosk arm.	Screening	Dementia
Schrager, Justin D and Smith, L Shakiyla and Heron, Sheryl L and Houry, Debra	Academic Emergency Medicine	Does Stage of Change Predict Improved Intimate Partner Violence Outcomes Following an Emergency Department Intervention?	Emergency Department	3	2013	US	R	ED based kiosk screening and health information delivery on intimate partner violence associated with	Health Information / Screening	Domestic Violence / Home Safety

Author	Journal	Title	Setting	# Kiosks	Yr	Country	Access	Comments	Purpose	Condition
								participants taking protective action.		
Shields, Wendy C and McDonald, Eileen M and McKenzie, Lara and Wang, Mei-Cheng and Walker, Allen R and Gielen, Andrea C	Pediatric Emergency Care	Using the Pediatric Emergency Department to Deliver Tailored Safety Messages	Emergency Department	1	2013	US	R	Kiosk to deliver tailored safety messages to participants. Knowledge increased among participants using the kiosks.	Health Information	Pediatric Injuries
Smith, Sarah E and Ludwig, John T and Chinchilli, Vernon M and Mehta, Khanjan and Stoute, Jos{\'{e}} A	Telemedicine and e-Health	Use of Telemedicine to Diagnose Tinea in Kenyan Schoolchildren	Community	1	2013	KE	R	Telemedicine diagnosis of skin conditions via digital images taken by kiosk was possible.	Screening	Dermatolo gy
WILAMOWSKA, KATARZYNA and LE, THAI and DEMIRIS, GEORGE and THOMPSON, HILAIRE	CIN: Computers, Informatics, Nursing	Using Commercially Available Tools for Multifaceted Health Assessment	Retirement community	1	2013	US	R	Data integration from multiple sources was possible using freeware.	Telehealth	Various
Ackerman, Sara L and Tebb, Kathleen and Stein, John C and Frazee, Bradley W and Hendey, Gregory W and Schmidt, Laura A and Gonzales, Ralph	Social Science {\&} Medicine	Benefit or burden? A sociotechnical analysis of diagnostic computer kiosks in four California hospital emergency departments	Emergency Department	4	2012	US	R	Examination of the reasons for non-adoption of a kiosk to diagnose and expedite treatment of female UTI. The implementation failed to take into account the complexities of the ED work at each site.	Screening	UTI
Alpert, Bruce S	Blood Pressure Monitoring	Are kiosk blood pressure readings	Pharmacy	NA	2012	US	O	Only one out of seven companies	Clinical Measurements	Hypertensi on

Author	Journal	Title	Setting	# Kiosks	Yr	Country	Access	Comments	Purpose	Condition
		trustworthy?						contacted provided complete validation data for their automated BP Kiosk.		
Gilbert, Julie E and Howell, Doris and King, Susan and Sawka, Carol and Hughes, Erin and Angus, Helen and Dudgeon, Deborah	Journal of Pain and Symptom Management	Quality Improvement in Cancer Symptom Assessment and Control: The Provincial Palliative Care Integration Project (PPCIP)	Specialty Clinic	12	2012	CA	R	Symptom Screening for Cancer patients; Improvements were made in symptom screening, symptom control and functional assessment.	Clinical Measurements	Cancer
Haukoos, Jason S and Hopkins, Emily and Bender, Brooke and Al-Tayyib, Alia and Long, Jeremy and Harvey, Jeffrey and Irby, Jessica and Bakes, Katherine	Academic Emergency Medicine	Use of Kiosks and Patient Understanding of Opt-out and Opt-in Consent for Routine Rapid Human Immunodeficiency Virus Screening in the Emergency Department	Emergency Department	1	2012	US	R	Computerized kiosks were used to successfully perform nontargeted rapid HIV screening	Screening	HIV
Houle, Sherilyn K.D. and Chuck, Anderson W and Tsuyuki, Ross T	Journal of the American Pharmacists Association	Blood pressure kiosks for medication therapy management programs: Business opportunity for pharmacists	Pharmacy	341	2012	CA	O	Economic model for BP kiosks in pharmacies. Average of $12,270 income per pharmacy per year could be generated.	Clinical Measurements	Hypertension
Joshi, Ashish and Trout, Kate	GLOBAL HEALTH 2012, The First International	Heath Kiosks as an Equal Opportunity Resource for Better Health: A Systematic	Review	NA	2012	Various	O/R	Systematic Review of Health Kiosks. 32 studies	Review	Various

Author	Journal	Title	Setting	# Kiosks	Yr	Country	Access	Comments	Purpose	Condition
	{\ldots}	Review						included. Sustainable and accessible health kiosks would have a positive impact.		
KURODA, Masahiro	IEICE Transactions on Communications	Healthcare ICT for Temporary Housing Community in Disaster-Stricken Area	Support Center	2	2012	JP	R	Kiosk system for Health checks at a support center for victims of disasters	Clinical Measurements	Health and Wellbeing
Resnick, Helaine E and Ilagan, Perla R and Kaylor, Mary Beth and Mehling, Diane and Alwan, Majd	Telemedicine and e-Health	TEAhM�Technologies for Enhancing Access to Health Management: A Pilot Study of Community-Based Telehealth	Senior Center	2	2012	US	R	Kiosk for remote monitoring of BP in a senior center. The kiosk was embraced by clients and staff of the senior centers.	Telehealth	Hypertension
Shields, Wendy C and McDonald, Eileen M and Stepnitz, Rebecca and McKenzie, Lara T and Gielen, Andrea C	Pediatric Emergency Care	Dog Bites	Emergency Department	1	2012	US	R	Patient education about dog bites delivered via kiosk. Results showed that parents using the kiosk increased knowledge about dog bites.	Health Information	Dog bites
ANDERSEN, SUSAN and ANDERSEN, PER and YOUNGBLOOD, NORMAN "ED"	CIN: Computers, Informatics, Nursing	Multimedia Computerized Smoking Awareness Education for Low-Literacy Hispanics	Primary Care	1	2011	US	O	Education about Smoking awareness and cessation. Feedback showed method was acceptable.	Health Information	Smoking
Dirocco, Danae N and Day, Susan C	The American journal of managed care	Obtaining patient feedback at point of service using electronic kiosks.	Primary Care	1	2011	US	R	Collection of patient feedback at a primary care clinic. Was	Patient feedback	Various

Author	Journal	Title	Setting	# Kiosks	Yr	Country	Access	Comments	Purpose	Condition
								successful in obtaining feedback without disrupting workflow.		
Eaton, Charles B and Parker, Donna R and Borkan, Jeffrey and McMurray, Jerome and Roberts, Mary B and Lu, Bing and Goldman, Roberta and Ahern, David K	The Annals of Family Medicine	Translating Cholesterol Guidelines Into Primary Care Practice: A Multimodal Cluster Randomized Trial	Primary Care	15	2011	US	R	Heart Disease and Cholesterol Control Guideline education delivered via Kiosk	Health Information	Cardiovascular Disease
Hovey, Lauren and Kaylor, Mary Beth and Alwan, Majd and Resnick, Helaine E	Telemedicine and e-Health	Community-Based Telemonitoring for Hypertension Management: Practical Challenges and Potential Solutions	Senior Center	2	2011	US	R	Telemonitoring for Hypertension Management-Challenges and Solutions	Clinical Measurements	Hypertension
Leijon, Matti and Arvidsson, Daniel and Nilsen, Per and {Stark Ekman}, Diana and Carlfjord, Siw and Andersson, Agneta and Johansson, Anne Lie and Bendtsen, Preben	Journal of Medical Internet Research	Improvement of Physical Activity by a Kiosk-based Electronic Screening and Brief Intervention in Routine Primary Health Care: Patient-Initiated Versus Staff-Referred	Primary Care	28	2011	SE	O/R	Screening and guidance for increasing physical activity	Health Information	Health and Wellbeing
Lesselroth, Blake J and Holahan, Patricia J and Adams, Kathleen and Sullivan, Zhen Z and Church, Victoria L and Woods, Susan and Felder, Robert and Adams, Shawn and Dorr,	Informatics in primary care	Primary care provider perceptions and use of a novel medication reconciliation technology.	Primary Care	7	2011	US	R	Kiosk for patients to complete medical reconciliation.	Medication reconciliation	Medication

Author	Journal	Title	Setting	# Kiosks	Yr	Country	Access	Comments	Purpose	Condition
David A										
McIndoe, Robert Stephen	Ethical Issues and Security Monitoring Trends in Global Healthcare (Book)	Health Kiosk Technologies	Review	NA	2011	UK	O/R	This chapter examines the recent rise in the adoption and spread of kiosks in UK healthcare: The leading players in the healthcare market, their technologies, the main uses of the kiosks and software, including possible future developments into clinical care.	Review	Various
McMullen, Karen D and McConnaughy, Rozalynd P and Riley, Ruth A	Journal of consumer health on the Internet	Outreach to Improve Patient Education at South Carolina Free Medical Clinics.	Primary Care	2	2011	US	O	Provision of Health Information (Medline Plus) in two kiosks at two clinics	Health Information	Various
Price, Erika Leemann and MacKenzie, Thomas D. and Metlay, Joshua P. and Camargo, Carlos A. and Gonzales, Ralph	Patient Education and Counseling	A computerized education module improves patient knowledge and attitudes about appropriate antibiotic use for acute respiratory tract infections	Emergency Department	8	2011	US	R	Computerized education module about antibiotic use for respiratory infections	Health Information	Medicatio n
Putre, Laura	Hospitals {\&} health networks	Technology. No line, no waiting; electronic kiosks speed up patient registration.	Hospital	38	2011	US	R	Kiosks for patient registration and check-in.	Patient registration	Various

Author	Journal	Title	Setting	# Kiosks	Yr	Country	Access	Comments	Purpose	Condition
Saifu, Hemen N and Shamouelian, Albert and Davis, Lisa G and Santana-Rios, Elizabeth and Goetz, Matthew Bidwell and Asch, Steven M and Sun, Benjamin C	Journal of Telemedicine and Telecare	Impact of a kiosk educational module on HIV screening rates and patient knowledge	Specialty Clinic	1	2011	US	R	Kiosk delivered educational module for HIV Screening. Kiosk use increased overall patient knowledge about HIV testing.	Health Information	HIV
Scribano, Philip V and Stevens, Jack and Marshall, Jessica and Gleason, Erica and Kelleher, Kelly J	Pediatric emergency care	Feasibility of computerized screening for intimate partner violence in a pediatric emergency department.	Emergency Department	2	2011	US	R	Home safety screening for Intimate Partner Violence	Screening	Domestic Violence / Home Safety
Thompson, Hilaire J. and Demiris, George and Rue, Tessa and Shatil, Evelyn and Wilamowska, Katarzyna and Zaslavsky, Oleg and Reeder, Blaine	Telemedicine and e-Health	A Holistic Approach to Assess Older Adults' Wellness Using e-Health Technologies	Retirement community	1	2011	US	R	Collection of clinical measurements: BP, HR, O2Sat, Blood Glucose	Telehealth	Various
Weaver, Nancy L and Nansel, Tonja R and Williams, Janice and Tse, Julia and Botello-Harbaum, Maria and Willson, Katherine	Translational Behavioral Medicine	Reach of a kiosk-based pediatric injury prevention program	Primary Care	5	2011	US	R	Education to reduce childhood injuries.	Health Information	Pediatric Injuries
{A. Paula Cupertino} and {Kimber Richter} and {Lisa Sanderson Cox} and {Susan Garrett} and {Rigoberto Ramirez} and {Fernando Mujica} and {Edward F. Ellerbeck}	Journal of Health Care for the Poor and Underserved	Feasibility of a Spanish/English Computerized Decision Aid to Facilitate Smoking Cessation Efforts in Underserved Communities	Multiple	5	2010	US	R	Bilingual Kiosk to give decision aid in smoking cessation	Health Information	Smoking
Gance-Cleveland,	Journal for	Evaluation of	Primary	1	2010	US	R	Kiosk for	Clinical	Childhood

Author	Journal	Title	Setting	# Kiosks	Yr	Country	Access	Comments	Purpose	Condition
Bonnie and Gilbert, Lynn H. and Kopanos, Taynin and Gilbert, Kevin C.	Specialists in Pediatric Nursing	technology to identify and assess overweight children and adolescents	Care					collection of Height, Weight, BP and calculation of BMI to identify childhood obesity	Measurements	obesity
Goldstein, Jack	Clinical Orthopaedics and Related Research{\text registered}	Private Practice Outcomes: Validated Outcomes Data Collection in Private Practice	Specialty Clinic	1	2010	US	R	Collection of Orthopedic Patients Outcomes data via interactive Kiosk	Patient Outcomes Data	Orthopedics
Leeman-Castillo, Bonnie and Beaty, Brenda and Raghunath, Silvia and Steiner, John and Bull, Sheana	American Journal of Public Health	LUCHAR: Using Computer Technology to Battle Heart Disease Among Latinos	Multiple	5	2010	US	R	Cardiovascular Disease Health Education through kiosks	Health Information	Cardiovascular Disease
Lowe, Charles and Cummin, Debbie	Journal of Telemedicine and Telecare	The use of kiosk technology in general practice	Primary Care	18	2010	UK	R	Qualitative Study: Interview with practice managers GP's and patients where kiosks had been used.	Clinical Measurements	Various
Pendleton, Brian F and Schrop, Susan Labuda and Ritter, Christian and Kinion, Elizabeth S and McCord, Gary and {Cray Jr.}, James J and Costa, Anthony J	FAMILY MEDICINE	Underserved Patients' Choice of Kiosk-based Preventive Health Information	Primary Care	6	2010	US	O	Kiosks installed in 6 clinics to provide health education for patients in waiting room	Health Information	Health and Wellbeing
Sano, Mary and Egelko, Susan and Ferris, Steven and Kaye, Jeffrey and Hayes, Tamara L and	Alzheimer Disease {\&} Associated Disorders	Pilot Study to Show the Feasibility of a Multicenter Trial of Home-based Assessment of	Home	18	2010	US	R	RCT of Home-based Alzheimer assessment using Mail and Phone, IVR, and Kiosk.	Cognitive assessments	Dementia

Author	Journal	Title	Setting	# Kiosks	Yr	Country	Access	Comments	Purpose	Condition
Mundt, James C and Donohue, Michael and Walter, Sarah and Sun, Shelly and Sauceda-Cerda, Luis		People Over 75 Years Old								
Sun, Benjamin C and Knapp, Herschel and Shamouelian, Albert and Golden, Joya and Goetz, Matthew Bidwell and Asch, Steven M	Journal of Telemedicine and Telecare	Effect of an education kiosk on patient knowledge about rapid HIV screening	Specialty Clinic	1	2010	US	O	Health Education about Rapid HIV screening	Health Information	HIV
Teolis, Marilyn G	Journal of consumer health on the Internet	A MedlinePlus Kiosk Promoting Health Literacy.	Primary Care	1	2010	US	O	Health Information Kiosk offering tutorials on using Medline Plus	Health Information	Various
Trepka, Mary Jo and Newman, Frederick L and Huffman, Fatma G and Dixon, Zisca	Journal of Nutrition Education and Behavior	Food Safety Education Using an Interactive Multimedia Kiosk in a WIC Setting: Correlates of Client Satisfaction and Practical Issues	Specialty Clinic	1	2010	US	R	Food Safety education with an interactive multimedia Kiosk	Health Information	Food Safety
Harlin, Stephen L and Harlin, Ryan D and Sherman, Thomas I and Rozsas, Courtney M and Shafqat, M Shuja and Meyers, William	Ostomy/wound management	Using a structured, computer-administered questionnaire for evaluating health-related QOL with chronic lower extremity wounds.	Specialty Clinic	1	2009	US	R	Administered Quality of Life survey for patients with chronic wounds	Survey Administration	Wounds
Jones, Ray	International Journal of Environmental Research and Public Health	The Role of Health Kiosks in 2009: Literature and Informant Review	Review	NA	2009	Various	O/R	Literature and Informant Review of use of Health Kiosks in 2009	Review	Various
Joshi, Ashish and	Health	Prospective tracking	Emergency	1 (?)	2009	US	R	Health Education	Health	Asthma

Author	Journal	Title	Setting	# Kiosks	Yr	Country	Access	Comments	Purpose	Condition
Weng, Wenjing and Lichenstein, Richard and Arora, Mohit and Sears, Andrew	Informatics Journal	of a pediatric emergency department e-kiosk to deliver asthma education	Department					about Asthma in Pediatric Patients	Information	
Lesselroth, Blake and Adams, Shawn and Felder, Robert and Dorr, David A and Cauthers, Phillip and Church, Victoria and Douglas, David	Joint Commission journal on quality and patient safety	Using consumer-based kiosk technology to improve and standardize medication reconciliation in a specialty care setting.	Specialty Clinic	1	2009	US	R	Kiosk system for medication history and reconciliation. Patients with little or no computer experience found it easy to use.	Medication reconciliation	Medication
Lesselroth, Blake J and Felder, Robert S and Adams, Shawn M and Cauthers, Phillip D and Dorr, David A and Wong, Gordon J and Douglas, David M	Journal of the American Medical Informatics Association	Design and Implementation of a Medication Reconciliation Kiosk: the Automated Patient History Intake Device (APHID)	Primary Care	4	2009	US	R	Kiosk system for medication history and reconciliation. Patients with little or no computer experience found it easy to use.	Medication reconciliation	Medication
Matthews, Paul H and Darbisi, Carolina and Sandmann, Lorilee and Galen, Robert and Rubin, Donald	Journal of Immigrant and Minority Health	Disseminating health information and diabetes care for Latinos via electronic information kiosks	Multiple	2	2009	US	O	Health Information about diabetes. Patients found system helpful and useful.	Health Information	Diabetes
